# ICAM-1 on Breast Cancer Cells Suppresses Lung Metastasis but Is Dispensable for Tumor Growth and Killing by Cytotoxic T Cells

**DOI:** 10.3389/fimmu.2022.849701

**Published:** 2022-07-11

**Authors:** Ofer Regev, Marina Kizner, Francesco Roncato, Maya Dadiani, Massimo Saini, Francesc Castro-Giner, Olga Yajuk, Stav Kozlovski, Nehora Levi, Yoseph Addadi, Ofra Golani, Shifra Ben-Dor, Zvi Granot, Nicola Aceto, Ronen Alon

**Affiliations:** ^1^Department of Immunology, Weizmann Institute of Science, Rehovot, Israel; ^2^Cancer Research Center, Sheba Medical Center, Ramat-Gan, Israel; ^3^Department of Biology, Institute of Molecular Health Sciences, ETH Zurich, Zurich, Switzerland; ^4^Department of Developmental Biology and Cancer Research, Institute for Medical Research Israel-Canada, Hebrew University Medical School, Jerusalem, Israel; ^5^Life Sciences Core Facilities, Weizmann Institute of Science, Rehovot, Israel

**Keywords:** adhesion, killing, metastasis, integrins, neutrophils, vasculature

## Abstract

Breast tumors and their derived circulating cancer cells express the leukocyte β_2_ integrin ligand Intercellular adhesion molecule 1 (ICAM-1). We found that elevated ICAM-1 expression in breast cancer cells results in a favorable outcome and prolonged survival of breast cancer patients. We therefore assessed the direct *in vivo* contribution of ICAM-1 expressed by breast cancer cells to breast tumorigenesis and lung metastasis in syngeneic immunocompetent mice hosts using spontaneous and experimental models of the lung metastasis of the C57BL/6-derived E0771 cell line, a luminal B breast cancer subtype. Notably, the presence of ICAM-1 on E0771 did not alter tumor growth or the leukocyte composition in the tumor microenvironment. Interestingly, the elimination of Tregs led to the rapid killing of primary tumor cells independently of tumor ICAM-1 expression. The *in vivo* elimination of a primary E0771 tumor expressing the ovalbumin (OVA) model neoantigen by the OVA-specific OVA-tcr-I mice (OT-I) transgenic cytotoxic T lymphocytes (CTLs) also took place normally in the absence of ICAM-1 expression by E0771 breast cancer target cells. The whole lung imaging of these cells by light sheet microscopy (LSM) revealed that both Wild type (WT)- and ICAM-1-deficient E0771 cells were equally disseminated from resected tumors and accumulated inside the lung vasculature at similar magnitudes. ICAM-1-deficient breast cancer cells developed, however, much larger metastatic lesions than their control counterparts. Strikingly, the vast majority of these cells gave rise to intravascular tumor colonies both in spontaneous and experimental metastasis models. In the latter model, ICAM-1 expressing E0771- but not their ICAM-1-deficient counterparts were highly susceptible to elimination by neutrophils adoptively transferred from E0771 tumor-bearing donor mice. *Ex vivo*, neutrophils derived from tumor-bearing mice also killed cultured E0771 cells *via* ICAM-1-dependent interactions. Collectively, our results are a first indication that ICAM-1 expressed by metastatic breast cancer cells that expand inside the lung vasculature is involved in innate rather than in adaptive cancer cell killing. This is also a first indication that the breast tumor expression of ICAM-1 is not required for CTL-mediated killing but can function as a suppressor of intravascular breast cancer metastasis to lungs.

## Introduction

ICAM-1, also known as CD54, is a cell surface glycoprotein that is expressed primarily on endothelial cells and the cells of the immune system (1, 2). This immunoglobulin superfamily member binds to the integrins of the β2 family such as Lymphocyte function-associated antigen 1 (LFA-1) [αLβ2- LFA-1 integrin subunits (αLβ2), αL and β2 integrin subunits (CD11A/CD18)] and Mac-1 (αMβ2, CD11b/CD18) (3). Upon stimulation by primary cytokines like interleukin-1β (IL-1β) and the tumor necrosis factor (TNF), ICAM-1 is *de novo* transcribed on these cells. When activated by chemokines, leukocytes use their LFA-1 to bind endothelial ICAM-1 with high affinity, a prerequisite for their transmigration into tissues (4). ICAM-1 also serves as the entry receptor for human rhinovirus (HRV) on various epithelial cells (5).

ICAM-1 is expressed on the surface of several malignant cells and may thus contribute to both cancer growth and cancer immunosurveillance by adaptive and non-adaptive immune arms (6, 7). For instance, tumor-associated macrophages (TAMs) and neutrophils (TANs) may use their LFA-1 and Mac-1 to bind ICAM-1 on primary tumor cells and thereby facilitate tumor growth and angiogenesis. The invasiveness of breast cancer cells has been positively correlated with the expression of ICAM-1 (8). ICAM-1 on the human MDA-MB-231 breast cancer cell line was also implicated in circulating tumor cell (CTC) cluster formation, intravascular cell aggregation and increased metastatic potential (9, 10). In some settings, neutrophil–cancer cell interactions mediated by neutrophil LFA-1 and cancer ICAM-1 were shown to facilitate breast cancer metastasis (11). On the other hand, the massive killing of CTCs by macrophages (12) takes place in the liver and spleen (13) during metastasis, and the CTCs entrapped by lung capillaries are susceptible to direct killing by Natural killer (NK) cells and neutrophils (14–16). Whether ICAM-1 expressed by CTCs contributes to these innate immunity processes is currently unknown.

The adaptive immune arm was also suggested to use cancer cell–expressed ICAM-1 for tumor elimination (17). The productive engagement of ICAM-1 on target cells by CTLs was suggested to enhance the presentation of cognate peptide MHC-I complexes (pMHCs) to these killer lymphocytes (18). *In vitro*, tumor-specific LFA-1-expressing endogenous cytotoxic T lymphocytes (CTLs) were shown to kill tumor cells *via* a direct recognition of the tumor cell ICAM-1 (19). The engagement of tumor ICAM-1 and CTL LFA-1 can also facilitate cytokine production by CTLs (20). Nevertheless, the *in vivo* roles of ICAM-1 in breast cancer cell growth and metastasis are still poorly understood. Metastasis is the main cause of death in cancer patients. To study this complex process in an immunologically relevant context, researchers use various animal models of metastasis, with different experimental limitations. In the present study, in order to investigate the role of ICAM-1 in a breast cancer model of tumor growth, progression, and metastasis, we used both spontaneous and experimental metastasis models based on the metastatic, C57BL/6 derived, medullary breast adenocarcinoma E0771 cell line. This breast cancer line, originally isolated from spontaneous cancer in C57BL/6, spreads to multiple organs including the lungs (21, 22). We report that neither the growth of this breast cancer line in primary orthotopic tumors nor its susceptibility to killing by CTLs is affected by ICAM-1 deletion. Furthermore, whereas the ability of E0771 cells within primary breast tumors to disseminate and reach the lungs is ICAM-1 independent; once entrapped inside the lung vasculature, ICAM-1 deficient-breast cancer cells but not their control counterparts generate intravascular metastatic lesions. Our results therefore elude to a novel contribution of ICAM-1 to breast cancer cell killing by neutrophils inside the lung vasculature.

## Materials and Methods

### Survival Analysis of Breast Cancer Patients

Kaplan–Meier curves for relapse-free survival and overall survival were calculated for all subtypes of invasive breast carcinoma and for the luminal B subtype. Datasets were derived from Gene Expression Omnibus (GEO; http://www.ncbi.nlm.nih.gov/gds) and The European Genome-phenome Archive (EGA) (https://www.ebi.ac.uk/ega/) and were analyzed by a Kaplan–Meier plotter (23). The cutoff for high and low groups was set by using the auto-select best cutoff. All other settings were set to default. Luminal B patient analysis was based on the 2013 StGallen criteria (24). P-values were calculated using the log-rank test (Mantel–Cox test) and by the Cox proportional hazard ratio using 95% confidence interval.

### Western Blot

Immunoblotting was performed as described previously (25). The antiestrogen receptor-α antibody (Clone F-10, Cat. SC-8002) was used to detect the receptor in E0771 cells. Michigan Cancer Foundation-7 (MCF-7) cells were used as a positive control. Glyceraldehyde-3-Phosphate Dehydrogenase (GAPDH) was used as a loading control.

### Murine Cell Lines

Murine breast adenocarcinoma cells (E0771) were purchased from CH3 Biosystems (Stock Keeping Unit (SKU): 94A001) and grown in Dulbecco's Modified Eagle Medium (DMEM) supplemented with 10% fetal bovine serum (FBS), 1-mM sodium pyruvate, 10-mM HEPES, and 1% penicillin–streptomycin–amphotericin B solution. G-CSF-overexpressing B16-F10 cells (26) were a generous gift of Dr. Asaph Spiegel from the Robert A. Weinberg lab at the Whitehead Institute for Biomedical Research, Cambridge, Massachusetts, and were grown in DMEM supplemented with 10% FBS and 1% penicillin–streptomycin–amphotericin B solution. 4T1 cells were obtained from American Type Culture Collection (ATCC) and grown in RPMI 1640 supplemented with 10% FBS and 1% penicillin–streptomycin–amphotericin B solution. Cell lines were passaged up to 10 times and were periodically validated as negative for mycoplasma. Cell cultures were kept at 37°C in a humidified incubator in the presence of 5% CO_2_. Murine CD8 effector T cells were generated from T cells isolated from the spleens of OT-I mice. Spleen-derived lymphocytes were stimulated *in vitro* with SIINFEKL-loaded syngeneic DCs and expanded in Interleukin-2 (IL-2)-rich media as described (27).

### CRISPR/Cas9 Gene Editing–Mediated Targeting of ICAM-1

Clustered regularly interspaced short palindromic repeats (CRISPR) guides were chosen using several design tools, including: the Massachusetts Institute of Technology (MIT) CRISPR design tool (28) and Single guide RNA (sgRNA) Designer, Rule set 2 (29), in the Benchling implementations (www.benchling.com), SSC (30), and sgRNAscorer (31), in their websites. E0771 cells were transfected by electroporation in Opti-Minimum Essential Media (MEM) media with the Cas9 enzyme (Cat. 1081058, Integrated DNA Technologies (IDT)), two guide RNA oligoes, and tracer RNA (Cat. 1073189, IDT). The sequences of the guide RNA oligos were GCGCGAGGTTTCCCGGAAAG (Guide 1) and AGGGATCACAACGGTGACCA (Guide 2). Cells were incubated for 72 h after transfection and were stained and sorted by fluorescence-activated cell sorting (FACS) for ICAM-1-positive and -negative expressing cells.

### Analysis of Cell Surface Molecules

For the analysis of cell surface molecule expression, E0771 cells were stained in an FACS buffer [Phosphate-buffered saline (PBS) supplemented with 2% bovine serum albumin (BSA) and 5-mM Ethylenediaminetetraacetic acid (EDTA)] with monoclonal antibodies specific to ICAM-1 (Allophycocyanin (APC) conjugated, clone YN/1.7.4, Cat. 116119, Biolegend); ICAM-2 (Alexa 647 conjugated, clone 3C4 (MIC2/4), Cat. 105611, Biolegend); CD47 (APC conjugated, clone miap301, Cat. 127513, Biolegend); Integrin β1 (APC conjugated, clone HMB1-1, Cat. 102215 Biolegend); CXCR4 (PE conjugated, clone 1.276F12, Cat. 146505, Biolegend); MHC-I (H2K-b) (APC-conjugated, Clone- AF6-88.5, Cat. 116517, Biolegend), and to SIINFEKL bound to H2K-b [Clone 25-D1.16 (32), Cat. 12-5743-82, Thermo Fisher]. Background stainings were determined with matched fluorescence-labeled isotype control monoclonal antibodies (mAbs). P-selectin ligand staining was performed with P-selectin fusion protein as previously described (33). BM neutrophils were stained with an anti-Ly6G antibody (APC/Cy7-conjugated, clone 1A8, Cat. 127623, Biolegend). Cell surface staining was analyzed in a CytoFLEX flow cytometer (Beckman Coulter).

### Orthotopic Tumor Experiments

E0771 cells were used as a syngeneic model for orthotopic breast cancer growth, spontaneous metastasis, and experimental metastasis in wild-type C57BL/6 mice (21, 22). Mice were maintained in a pathogen-free facility, and all animal procedures were approved by the Animal Care and Use Committee of the Weizmann Institute of Science. Orthotopic E0771 breast cancer lesions were generated by inoculating the mammary fat pad of 8–12-week-old recipient female mice with 1 × 10^3^ E0771 cells (suspended in 50 μl of Matrigel^®^ Matrix diluted 2-fold in PBS). The tumor size was assessed throughout the duration of the experiment by the caliper measurements (34) of length (L) and width (W), and the tumor volume (V) was calculated using the formula: V = (L × W × W)/2. For spontaneous metastasis, 3 × 10^5^ tumor cells (suspended in Matrigel^®^, 1:1 in 50 μl of PBS solution) were implanted in the mammary fat pad of recipient female mice. Approximately 2–3 weeks later, when the tumor diameter reached approximately 1.5 cm, tumors were surgically removed. Then, 2–3 weeks later, mice were sacrificed by administration of sodium pentobarbital (200 mg/kg), and lungs were harvested and prepared for LSM or FACS analysis as described in other sections.

### *In Vitro* Tumor Cell Growth and Growth Arrest Analysis

Control and ICAM-1 knockout (KO) E0771 cells were seeded at 20,000 cells per well in a 6-well tissue culture plate. To determine their growth, cells were trypsinized, suspended, and counted by flow cytometer. To determine etoposide-induced growth arrest, E0771 cells were plated at 10,000 cells per well in a 96-well tissue culture plate. Approximately 1 day later, etoposide was added to the culture media at various concentrations. Then, 48 h later, the number of cells in each well was determined by flow cytometry and growth inhibition was calculated as


$$\left(1-\;\frac{\#\;etoposide\;treated\;cells}{\#\;untreated\;cells}\;\right)x\;100.\text{ Each treatment was performed in triplicates}.$$

### Tumor Immunohistochemistry

A total of 10^3^ control or ICAM-1 KO E0771 cells (each suspended in Matrigel, 1:1 in PBS) were implanted in the mammary fat pad and 10 days later, tumors were harvested and fixed O.N. in 4% PFA/PBS solution. Paraffin embedding and haematoxylin and eosin stain (H&E) staining of 5-µm-thin sections were performed in the histology core unit of the Weizmann Institute of Science. Sections were stained according to standard protocols for ICAM-1 IHC (Cat 550547, Biolegend) and for 4′,6-diamidino-2-phenylindole (DAPI). Microscopy-recorded sections were digitalized using a Panoramic SCAN II (3DHISTECH) and analyzed using CaseViewer software (3DHISTECH).

### Flow Cytometry Analysis of Cell Populations in the Primary Tumor, Tumor-Draining Lymph Nodes, and Spleen

A total of 10^3^ or 3 × 10^5^ control or ICAM-1 KO E0771 cells were implanted in the mammary fat pad as described in previous sections. Approximately 10 days later, tumors were harvested, minced and incubated in RPMI-1640 containing collagenase A (1.5 mg/ml) and DNase I (20 µg/ml) at 37°C for 45 min (35). Tumor cell suspensions were transferred through a 100-µm cell strainer and centrifuged at 0.2 × g for 5 min at 4°C. Red blood cells (RBCs) were subsequently lysed with and an RBC lysis buffer (Sigma-Aldrich, Rehovot, Israel, Cat. R7757). The remaining cells were resuspended in an ice-cold FACS buffer (PBS with 1% BSA, 0.1% sodium azide and 5-mM EDTA), filtered through a 100-µm strainer, stained according to standard FAC protocols with the cocktails of mAbs to specific cellular markers, and analyzed using a CytoFLEX flow cytometer. In parallel experiments, tumor-draining lymph nodes and spleen were harvested and cell suspensions were prepared, stained and analyzed as above.

### T Regulatory Cell Depletion Experiments

10^3^ control or ICAM-1 KO E0771 cells (suspended in Matrigel, 1:1 in PBS) were implanted in the mammary fat pad of FOXP3-Diphtheria toxin receptor-Green fluorescent protein (DTR-GFP) mice (36). From day 5 to day 13, a diphtheria toxin (DTx) (50-µg/kg body weight, Cat. D0564, Sigma-Aldrich) was injected Intraperitoneal (i.p) every 2 days. The E0771 tumor size was measured on days 11, 13, and 15. Tumor-draining lymph nodes were harvested on day 15, single-cell suspensions were prepared, and their T regulatory cell (Treg) content was determined by the flow cytometry of GFP-positive cells.

### *In Vitro* and *In Vivo* Killing of OVA-Expressing E0771 Cells

Control and ICAM-1 KO E0771 cells were stably transduced with an OVA-mCherry-encoding construct cloned in the pHR OVA/p2a/mCherry-CaaX lentiviral vector (Addgene, cat. 113030). Cells expressing the mCherry reporter were sorted and their expression of the OVA SIINFEKL peptide complexed with cell surface MHC-I (H-2Kb) was measured by 25-D1.16 mAb staining (32)). Control or ICAM-1 KO was seeded in a 96-well plate and cultured O.N. OT-1 effector CTLs were labeled with Carboxyfluorescein succinimidyl ester (CFSE) and overlaid on the tumor cells at different effector to target ratios. One day later, the number of viable E0771 cancer cells was determined by FACS. For *in vivo* killing analysis, 10^3^ control or ICAM-1 KO E0771 cells (suspended in Matrigel, 1:1 in PBS) were implanted in the mammary fat pad. Approximately 3 days later, 10^7^ effector OT-I CTLs were injected intravenous (i.v) and the tumor size was measured 12, 14, and 16 days later.

### *In Vitro* CTL Adhesion to Monolayers of E0771 Breast Cancer Cells

1 × 10^5^ control or ICAM-1 KO E0771 cells were seeded on a μ-SlideVI0.4 ibiTreat (ibidi) previously coated with 5 μg/ml fibronectin (Cat. #F1141, Sigma). A day later, the ibidi slide was connected to a microfluidic flow system. OT-1 CTLs were prepared as described above and perfused in binding medium (Hank’s balanced-salt solution containing 2-mg/ml BSA and 10-mM HEPES pH 7.4, supplemented with 1-mM CaCl_2_ and 1-mM MgCl_2_) into the chamber and allowed to settle on the different E0771 cell monolayers for 4 min in the absence of shear flow. Adherent CTLs were subsequently subjected to progressively increasing shear stresses (started at 5 dyn/cm^2^ and increased to 30 dyn/cm^2^ with 5-s-long intervals as described (37)). Real time imaging was performed utilizing a phase contrast IX83 Inverted Microscope (Olympus) and CTLs that remained firmly adherent to the E0771 monolayers at the end of the detachment assay were counted using cellSens Dimension v1.16 software (Olympus). The fractions of adherent CTLs out of originally settled CTLs were determined in nine fields of view (664 × 664 μm; 324 × 324 nm/pixel).

### *In Vitro* Neutrophil-Mediated Killing of E0771 Cells

Primary tumors were generated by orthotopic injections of 0.5 × 10^6^ E0771 cells into the mammary fat pad. Neutrophils were purified 3 weeks later as described before (38). In brief, whole blood was collected by cardiac puncture using a heparinized (Sigma) syringe. The blood was subjected to a discontinuous Histopaque (Sigma) gradient (1.077 and 1.119), and normal-density neutrophils were collected as described (15). Luciferase-containing E0771 cells (10,000/well) were plated in RPMI supplemented with 2% FBS. Approximately 4 h later, purified neutrophils (100,000/well) were added to the plated tumor cells and cocultured overnight with the medium. Following overnight incubation, luciferase activity was measured using a Tecan F200 microplate luminescence reader as described (15).

### CTC Analysis in Blood

Orthotopic 4T1 breast cancer lesions were generated in 8–10-week-old NSG females by injection of 0.5 × 10^6^ 4T1-GFP cells into the mammary fat pad as described (39). A blood draw for CTC analysis was performed 3 weeks after implantation through cardiac puncture and processed immediately on a Parsortix microfluidic device (39). Cancer cell-white blood cell (WBC) conjugates were detected by GFP and anti-CD45 staining as described (39).

### Light Sheet Microscopy of Lungs

For spontaneous metastasis, 3 × 10^5^ tumor cells (suspended in Matrigel^®^, 1:1 in 50 μl of PBS solution) were implanted in the mammary fat pad of recipient female mice. CD31-positive vessels were labeled 15 min before mice were sacrificed by an intravenous injection of 6 µg of Alexa-647 conjugated anti-CD31 mAb (Cat. 102515, clone MEC13.3). Immediately after the sacrifice, mice were transcardially perfused with PBS and the lungs were inflated *via* the trachea with low-gelling agarose (Cat. A9045, Sigma-Aldrich), fixed with paraformaldehyde (4% in PBS) for 2 h, dehydrated, and cleared using ethyl cinnamate as described (40, 41). Cleared lung lobes were imaged in an Ultramicroscope II (LaVision BioTec) operated by the ImspectorPro software (LaVision BioTec, Bielefeld, Germany) as described (41). For experimental metastasis, 1 × 10^4^ E0771 cells labeled with 10-µM (5-(and-6)-(((4-chloromethyl)benzoyl)amino)tetramethylrhodamine) (CMTMR) (Cat. C2927, Thermo Fisher Scientific) for 30 min were washed and injected in the retro-orbital sinus of recipient mice. CD31-positive vessels were labeled as described above and VCAM-1 positive vessels were labeled by intravenous injection of 5 µg of an Alexa-647 conjugated anti-VCAM-1 mAb (Cat. BD561612, clone 429 (MVCAM.A) BD). For nuclear staining, CMTMR-labeled E0771 cells were labeled with REDDOT1 (Cat. BTM-40060-T, NBT).

The three-dimensional rendering of LSM was performed *via* Imaris software (Oxford Instruments, Abingdon, UK). The surfaces of RFP or CMTMR-labeled tumor cells were created using volume (ranging from 1,000 to 200,000 μm^4^) and intensity (max of red fluorescent channel) as defining features to unequivocally separate them from back-ground signals. Each cell was individually segmented and its distance was measured with respect to the CD31-labeled blood vessels. The cell positions of intravascular were determined by this spatial analysis. For the neutrophil location, spots of Ly6G-647-labeled neutrophils were created based on the relative intensity. The distance between the centroids of tumor cells and neutrophils was determined using distance transformation. The distance between tumor cells and vessels was determined using the shortest distance calculation. For tumor nuclear staining, nuclei surfaces were reconstituted using intensity plots.

### Determination of Tumor Cells in Total Lung Cell Suspensions

CMTMR-labeled E0771 cells were resuspended in PBS and injected into the retro-orbital sinus of recipient mice. Mice were sacrificed and transcardially perfused with PBS, and the lungs were extracted, minced, and incubated in RPMI 1640 containing collagenase type 4 (1.5 mg/ml) and Dnase I (20 µg/ml) at 37°C for 40 min. Lung cell suspensions were transferred through a 1,000-µm cell strainer and centrifuged at 0.2 × g or 5 min at 4°C. RBCs were subsequently lysed, and cells were resuspended in an ice-cold FACS buffer, filtered through a 70-µm strainer and analyzed using a CytoFLEX flow cytometer. The CMTMR label was readily distinguishable from the background fluorescence of lung cells.

### Experimental Lung Metastasis and Histological Analysis

A total of 10^4^ E0771 cells were injected into the retro-orbital of recipient mice (41) and mice were sacrificed 14 days later, transcardially perfused with PBS, and the lungs were extracted and stored in 4% PFA for 24 h followed by incubation in 1% PFA for additional 24 h. The paraffin embedding and H&E staining of 5-µm-thin sections were performed by the histology core unit of the Weizmann Institute of Science. Sections were digitalized using a Panoramic SCAN II (3DHISTECH) and analyzed using CaseViewer software (3DHISTECH).

### Adoptive Transfer of Splenocytes From E0771 Tumor-Bearing Donor Mice

A total of 10 (3) WT E0771 cells (in 50 μl of Matrigel^®^ Matrix diluted 2-fold in PBS) were implanted in the mammary fat pad of C57BL/6 female donor mice. Approximately 14 days after tumor implantation, the spleens of the tumor-bearing donor mice or of naïve mice or of tumor-bearing mice that underwent neutrophil or CD8+ T-cell depletion (by the i.p. injection of the anti Ly6G or anti CD8 antibody 2 days before spleen isolation) were harvested. Notably, the neutrophil content in spleens isolated from WT and ICAM-1 null E0771 tumor-bearing mice was similar. Single-cell suspensions were prepared, and either whole splenocytes or splenocytes depleted of endogenous neutrophils or CD8 T cells were injected i.v. into recipient syngeneic mice into which 10^4^ control or ICAM-1 KO E0771 cells had been injected i.v. a day before. 13 days later, the lungs of the E0771 and splenocyte-injected recipient mice were harvested and processed for histological analysis as described above.

### Statistical Analysis

Data in graphs are represented as mean or mean ± standard error of the mean (SEM). Student’s two-tailed unpaired t-test or the Mann–Whitney two-tailed U test was used to determine the significance of the difference between the means of two groups. One- or two-way ANOVA tests were used to compare the means among three or more independent groups/categories. Significance was set to *p* < 0.05. The statistical details of experiments can be found in the figure legends.

## Results

### High ICAM-1 Expression in Human Breast Cancer Patients Correlates With Favorable Clinical Outcome

ICAM-1 is expressed by human and murine breast cancer cells (9). The analysis of breast cancer patient data (23) suggests that the expression of ICAM-1 on breast tumor cells is associated with longer relapse-free periods and a higher overall survival of breast cancer patients (**Supplementary Figure 1A**). The link between elevated ICAM-1 expression in breast cancer cells and the favorable outcome of breast cancer patients prompted us to assess the direct *in vivo* contribution of ICAM-1 expressed by breast cancer cells to breast tumorigenesis and lung metastasis in syngeneic immunocompetent mice hosts using E0771 as our breast cancer cell model.

### Knocking Out ICAM-1 in the C57BL/6 E0771 Breast Carcinoma Murine Line Does Not Affect Cancer Cell Growth

We first validated that the E0771 cells used throughout our study are HER2+ and express ER-α+ and can therefore be considered as luminal B breast cancer cells (**Supplementary Figure 1B**). We next verified that the relapse-free survival of luminal B cancer patients is significantly prolonged by an elevated ICAM-1 expression (**Supplementary Figure 1C**). To study the role of ICAM-1 in breast tumor progression and metastasis in a murine model, we deleted it from E0771 cells using CRISPR/Cas9 targeting (**Supplementary Figure 2**). Two RNA guides, 20 bp each, were designed to target a 178-bp segment that includes the initiator methionine in the first exon, common to all forms of the gene. By this strategy, the surface expression of the protein was abolished (**Supplementary Figure 2A**). The Cas9-targeted cells were sorted by FACs for either ICAM-1-negative cells (herein ICAM-1 KO E0771) or ICAM-1-positive cells expressing normal ICAM-1 levels [herein control E0771 cells (**Supplementary Figure 2B**)]. The deletion of the 17-bp segment was also verified by PCR (**Supplementary Figure 2C**). The characterization of other canonical adhesion molecules expressed by this breast cancer line revealed high expression levels of β1 integrins and low expression levels of the low affinity LFA-1 ligand ICAM-2 with a negligible expression of β2 integrins and P-selectin ligand implicated in cancer–cell platelet interactions (42) (**Supplementary Figure 3**). E0771 cells lack surface CXCR4, a widely expressed chemokine receptor involved in breast cancer survival and metastasis (43), but retains high surface levels of the “don’t eat me signal” CD47 (44), implicated in the escape of CTC from systemic macrophage and neutrophil-mediated phagocytosis (44) (**Supplementary Figure 3**). Importantly, the expression levels of these surface molecules were not affected by ICAM-1 deletion (**Supplementary Figure 3**), confirming the high selectivity of our CRISPR-targeted deletion strategy.

### Breast Cancer ICAM-1 Does Not Impair *In Vivo* Tumor Growth

**Figure 1:**
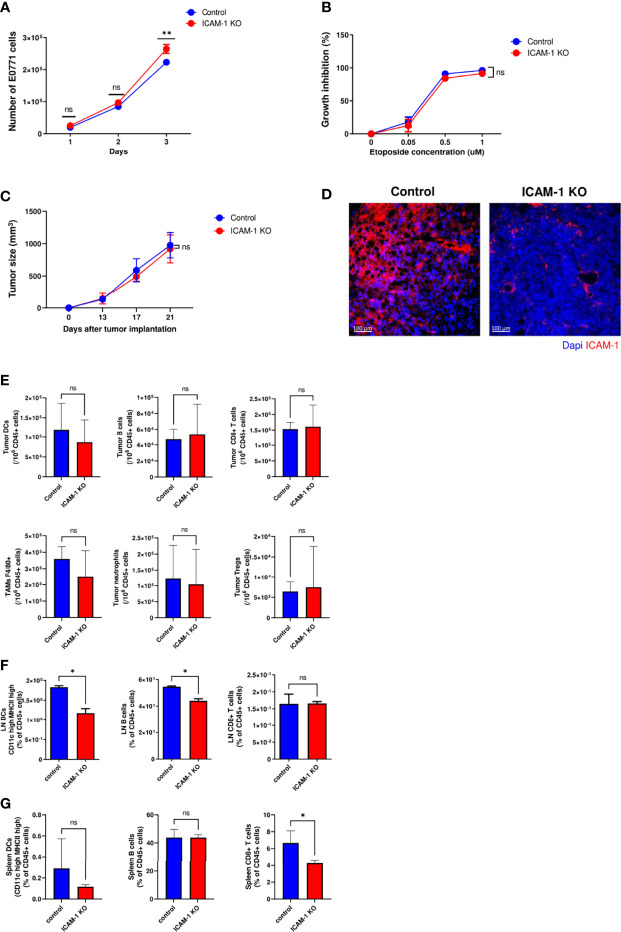
Tumor growth *in vitro* and *in vivo* is not affected by ICAM-1 expression on E0771 breast cancer cells. **(A)** Effect of E0771 ICAM-1 deletion on cell growth *in vitro*. 2 × 10^4^ control or ICAM-1 KO E0771 cells were seeded in 6-well plates in duplicates, and the growth curves of each experimental group were determined. The experiment was repeated twice. The results are an average of both experiments. **P< 0.005**. (B)** Effect of tumor ICAM-1 deletion on E0771 cell growth inhibition by the DNA damage agent etoposide. 1 × 10^4^ control or ICAM-1 KO E0771 cells were seeded in 96-well plates and exposed 1 day later to the indicated concentrations of etoposide. Approximately 48 h later, the cells were counted, and growth inhibition was determined and expressed as the percentage of triplicate values. The experiment was repeated twice. The results are an average of both experiments. The values are the mean ± SEM. **(C)** Effect of ICAM-1 deletion on E0771 growth *in vivo*. 1 × 10^3^ control or ICAM-1 KO E0771 cells suspended in Matrigel were implanted into the mammary fat pad of C57BL\6 female mice. The tumor volume was measured on the indicated days. The values are the mean ± SEM. n=5. **(D)** Primary tumors were implanted as in C and, 2 weeks later, were harvested and cut into 4-μm paraffin sections. Sections were immunostained for ICAM-1 and DAPI. A representative of 3–4 sections. **(E)** Myeloid and lymphoid compositions of the tumor microenvironment of primary control and ICAM-1 KO E0771 tumors. Tumors were implanted as in **(C)**. Approximately 14 days later, the tumors were excised for single-cell suspension preparation. The numbers of DCs (CD45+/MHC-II^hi^/CD11C^hi^), B cells (CD45+/CD19^hi^), CD8 T cells (CD45+/CD3+/CD8+), tumor-associated macrophages (CD45+/F4/80^hi^), neutrophils (CD45+/Ly6G+), and Tregs (CD45+/CD4+/CD25+/FOXP3+) were determined by FACS and were normalized to total CD45+ cells recovered from the tumors. N=7. **(F, G)** The numbers of DCs, B cells, and CD8+ T cells normalized to total CD45+ cells in the inguinal breast tumor-draining LNs **(F)** or the spleen **(G)**. Primary control and ICAM-1 KO E0771 tumors were implanted as in C, and 14 days later, the inguinal TdLNs and the spleen were harvested, and their content of DCs, B cells and CD8+ T cells was determined by FACs. The bars in E–G depict the average values ± SEM. n=3. *p< 0.05.

We next tested if ICAM-1 expression by E0771 cells affects the intrinsic growth of these breast cancer cells. To that end, 2 × 10^4^ control or ICAM-1 KO E0771 cells were seeded in 6-well plates and their proliferation was assessed. The early growth of both cell types was identical, but when cells approached confluency, ICAM-1 null cells grew slightly faster (**Figure 1A**). Knocking out ICAM-1 in E0771 did not affect the susceptibility of these cells to apoptotic signals induced by the potent DNA damage and cell cycle arrest inducer etoposide (**Figure 1B**).

Tumor growth in mice can be influenced by numerous paracrine signals. The common practice of cancer cell implantation involves a large number of cells that results in massive cell death and local inflammation that could mask any intrinsic or extrinsic effects resulting from ICAM-1 deletion. For this reason, we implanted a very low number of control and ICAM-1 KO E0771 cells embedded in Matrigel (45) in the mammary fat pad of syngeneic C57BL/6 female mice (i.e., 1 × 10^3^ cells) and followed tumor growth 13, 17, and 21 days after implantation (**Figure 1C**). As both control and ICAM-1 KO E0771 cells gave rise to similar tumors at all tested time points, we concluded that ICAM-1 expression by E0771 is dispensable for primary tumor growth *in vivo*. Notably, the implanted control E0771 cells maintained their surface ICAM-1 expression in this *in vivo* tumor growth model (**Figure 1D**).

Since all major types of hematopoietic CD45+ cells express the ICAM-1 binding LFA-1 and Mac-1 integrins (46–48), we next tested if the composition of these cells in the E0771 tumor microenvironment (TME) is affected by the absence of E0771-expressed ICAM-1 (**Figure 1E**). Consistent with the normal *in vivo* growth of ICAM-1 KO E0771 inside the mammary fat pad, the number of DCs, B cells, and TAMs recovered from orthotopic E0771 tumors was not affected by ICAM-1 deficiency in the implanted E0771 cells (**Figure 1E**). Importantly, the composition of naïve T cells and the accumulation of endogenous effector CD4+ and CD8+ T cells in the TME, as well as their expression of the immune checkpoint protein PD-1, were not significantly affected by the loss of E0771 ICAM-1 (**Supplementary Figure 4**). Interestingly, however, the accumulation of DCs and B cells, but not of CD8+ T cells, in the tumor-draining LNs (TdLNs) surrounding these tumors, was slightly reduced (**Figure 1F**) while their numbers in the spleens were unaffected (**Figure 1G**). These results indicate that breast cancer–expressed ICAM-1 may facilitate the egress of tumor-associated DCs from the tumor *via* lymphatics to nearby LNs and may consequently also enhance B-cell entry into these LNs.

### Treg Depletion and Tumor Vaccination Eliminate a Primary E0771 Breast Tumor Independently of ICAM-1 Expression on E0771 Cell Targets

**Figure 2:**
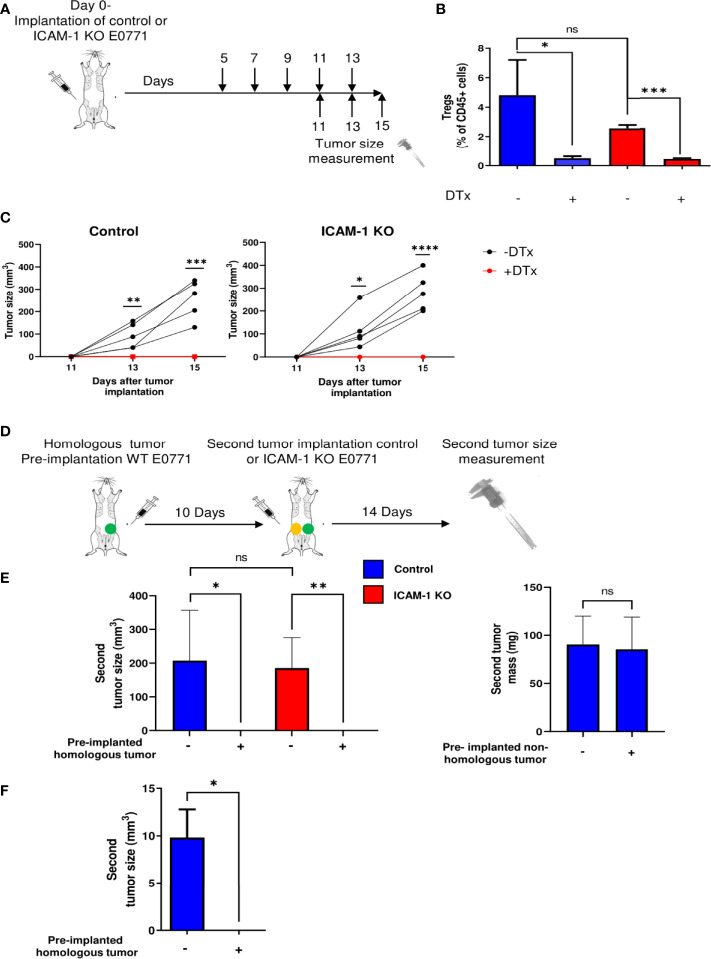
Treg depletion and homologous tumor preimplantation eliminate primary E0771 tumors independently of ICAM-1 expression on the breast cancer cells. **(A)** A scheme depicting the experiment. 1 × 10^3^ control or ICAM-1 KO E0771 cells were implanted in the mammary fat pad of C57BL/6 mice. DTx was injected every 2 days at the indicated time points, and the tumor size was measured on the indicated days. **(B)** Treg depletion by DTx injections was validated on day 17 in the TdLNs of mice implanted with control or the ICAM-1 KO E0771 cells. n=3. *p<0.05; ***p<0.0005; **(C)** The graphs depict the tumor size in the differently treated mice at the indicated time points. Black: untreated mice. Red: DTx-treated mice. n=5. * *p*<0.05; **p<0.005; ***p<0.0005; ****p<0.0001. At all time points, no significant differences were found between control and ICAM-1 KO E0771 tumor sizes. **(D)** The experimental scheme: a WT E0771 tumor/Matrigel or a Matrigel suspension was pre-implanted in the left mammary fat pad (green-filled circle). Approximately 10 days later, a second primary tumor composed of either control or ICAM-1 KO E0771 cells (in Matrigel) was implanted in the right mammary fat pad (yellow-filled circle), and 14 days later, the size of the right tumor was measured**. (E)** The mean volume of the second primary tumors is depicted for the four experimental groups. n=5. The bars depict the average values ± range of either the tumor volume (left panel) or mass (right panel). *p<0.05; ***p<0.0005. **(F)** A primary B16-G-CSF tumor (1 × 10^3^ cells) or control Matrigel was implanted in the left mammary fat pad. Approximately 10 days later, a primary tumor of control E0771 cells was implanted in the right mammary fat pad as in **(E)** Approximately 12 days later, the E0771 tumor weight was measured. The bars depict the mean values ± SEM. n=3. *p<0.05.

Tregs in the TME repress the ability of endogenous tumor-specific CTLs to eradicate primary tumors (49). To assess if this cytolytic activity of endogenous CTLs triggered by the elimination of Tregs requires the presence of ICAM-1 on E0771 tumor cells, we implanted primary E0771 tumors in syngeneic FOXP3-DTR-GFP mice that express a diphtheria toxin receptor selectively on FOXP3-positive Treg cells (50) and used a DTx to effectively deplete Tregs from the tumor-implanted mice (**Figures 2A** **, B**). When control or ICAM-1 KO E0771 cells were similarly implanted in the mammary fat pad of FOXP3-DTR-GFP recipients subjected to Treg depletion, both control and ICAM-1 KO E0771 tumors were eradicated to similar extents (**Figure 2C**). Thus, the presence of ICAM-1 on E0771 cells is not required for the CTL-mediated eradication of these cells *in vivo* triggered by Treg depletion. To further substantiate these results, we established another model of primary tumor killing by endogenous T cells. Instead of Treg depletion, we installed an E0771 tumor in a distal mammary fat pad (i.e., left mammary fat pad) 10 days before either control or ICAM-1 KO E0771 cells were implanted in the right mammary fat pad (**Figure 2D**). Notably, the presence of a distal primary E0771 tumor but not of an irrelevant distal tumor (e.g., B16 melanoma (16)) totally eliminated the growth of the second primary E0771 tumor but did so irrespectively of ICAM-1 expression by the second primary tumor (**Figure 2E**). Thus, the antitumor immune response experienced by E0771 cells inside a primary breast tumor elicited by E0771 cells implanted in a distal site does not depend on ICAM-1 expression on the tumor target. Notably, this anti-E0771 tumor killing by a distal E0771 tumor was FcR independent, and therefore ADCC or ADCP independent (51), since the distal E0771 tumor also efficiently eliminated the growth of the second primary E0771 tumor in FcR KO recipient mice (13) (**Figure 2F**). Taken together, these results suggest that the eradication of a primary E0771 breast tumor elicited by either Treg depletion or by a tumor vaccine is not facilitated by ICAM-1 expression on the breast cancer cell targets.

### Killing of OVA-Expressing E0771 Cells *In Vivo* by OT-I CTLs Is Also ICAM-1 Independent

**Figure 3:**
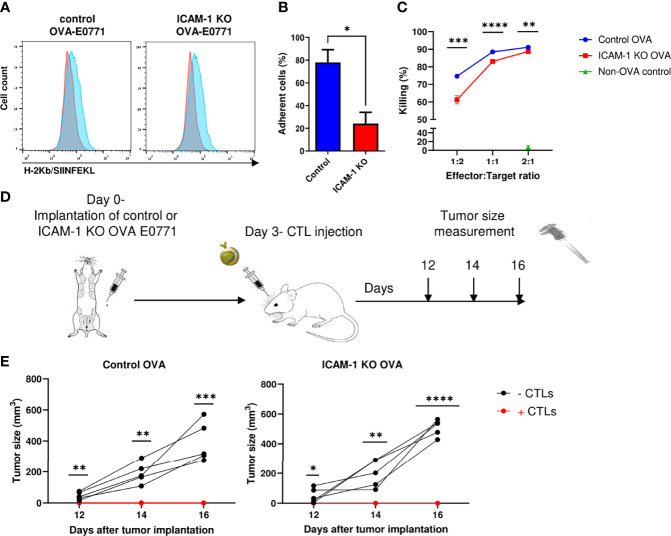
Killing of OVA-expressing breast cancer E0771 cells *in vivo* by *ex vivo* expanded OT-1 CTLs is ICAM-1 independent. **(A)** Expression of the OVA-derived SIINFEKL peptide complexed with H-2Kb on control and ICAM-1 KO E0771 cells infected with an OVA-mCherry encoding vector. The SIINFEKL/H-2Kb expression was detected with the 25-D1.16 mAb (32) and a secondary Ab (Blue). Staining with the control secondary Ab is shown in red. **(B)** ICAM-1 on E0771 cells is functionally adhesive. OT-1 effector CTLs were settled on the monolayers of control or ICAM-1 KO E0771 cells for 4 min and subsequently subjected to progressively increasing shear stresses as described in the *Materials and Methods* section. The fractions of CTLs that remained adherent to the monolayers at the highest shear stress were determined in three fields of view. *p<0.05. **(C)** The killing of control or ICAM-1 KO OVA- expressing E0771 cells or non-OVA expressing control E0771 cells by OT-I effector CTLs was tested *in vitro*. Tumor cells were seeded in triplicate in 96- well plates, and 24 h later, CTLs were added to the wells in different effectors to E0771 target ratios. The number of viable OVA-expressing tumor cells in the wells was counted 24 h later, and the percentage of killing was calculated as explained in the *Materials and Methods* section. n=4. **p<0.005; ***p<0.0005. ****p<0.0001 for control and ICAM-1 KO OVA E0771 groups. **(D)** A scheme depicting the experiment. Mice were implanted with control or ICAM-1 KO OVA expressing E0771 cells as in **Figure 1C**. Approximately 3 days later, 10^7^ of the OT-I effector CTLs were injected i.v. to each of the indicated experimental groups. **(E)** The tumor size was measured on days 12, 14, and 16 postimplantation. n=5. * *p*<0.05; **p<0.005; ***p<0.0005; ****p<0.0001.

To further dissect the involvement of tumor ICAM-1 in an antitumor immune response elicited by antitumor CTLs, we next tested if monoclonal effector CTLs specific for the OVA neoantigen introduced into E0771 cells can eliminate these cancer cells *in vitro* and *in vivo* in an ICAM-1-facilitated manner. To this end, we used OT-1 CTLs specific for the immunodominant ovalbumin (OVA)-derived peptide SIINFEKL presented by the MHC-I H-2Kb haplotype (27) abundantly expressed by both control and ICAM-1 KO E0771 cells (**Supplementary Figure 3**). Control and ICAM-1 KO E0771 cells were stably transfected with OVA, and the expression of SIINFEKL-bound MHC-1 (pMHC) complexes was analyzed with a specific antibody that recognizes the SIINFEKL/H-2Kb complex (52). Importantly, both control and ICAM-1 KO E0771 cells expressed comparable MHC-I (**Supplementary Figure 3**) and a similar content of SIINFEKL/H-2Kb complexes (**Figure 3A**). The adhesive function of ICAM-1 on E0771 cells was also verified since the monolayers of ICAM-1 KO E0771 cells devoid of cognate antigen supported weaker adhesions of *in vitro*-generated OT-I CTLs compared to control ICAM-1 expressing E0771 monolayers (**Figure 3B**). Furthermore, ICAM-1 KO OVA-expressing E0771 cells were slightly less efficiently killed by OT-I CTLs *in vitro* (**Figure 3C**).

We next tested if i.v.-injected effector OT-I CTLs can reach the mammary fat pad implanted with either control or ICAM-1 KO OVA- expressing E0771 cells and kill these target cells *in vivo* (**Figure 3D**). The primary breast tumors of either control or ICAM-1 KO OVA-expressing E0771 cells were implanted in syngeneic mice; 3 days later, OT-I CTLs were i.v.-injected into these mice, and the tumor size was monitored from day 12 to day 16 postinitial tumor implantation. The growth of the control OVA-expressing tumor was completely blocked by injected OT-I CTLs (**Figure 3E**). Consistently with our *in vitro* observations, the elimination of ICAM-1 KO OVA-expressing E0771 tumors by i.v.- injected OT-I CTLs was comparable to that of control OVA-expressing E0771 tumors (**Figure 3E**). Thus, the presence of ICAM-1 on OVA-expressing E0771 cells is fully dispensable for their *in vivo* elimination by adoptively transferred Ag-specific monoclonal CTLs (i.e., OT-I) as well as by endogenous polyclonal CTLs (**Figures 2C** **–E**).

### Breast Cancer ICAM-1 Suppresses Lung Metastasis in a Spontaneous Breast Tumor Model

**Figure 4:**
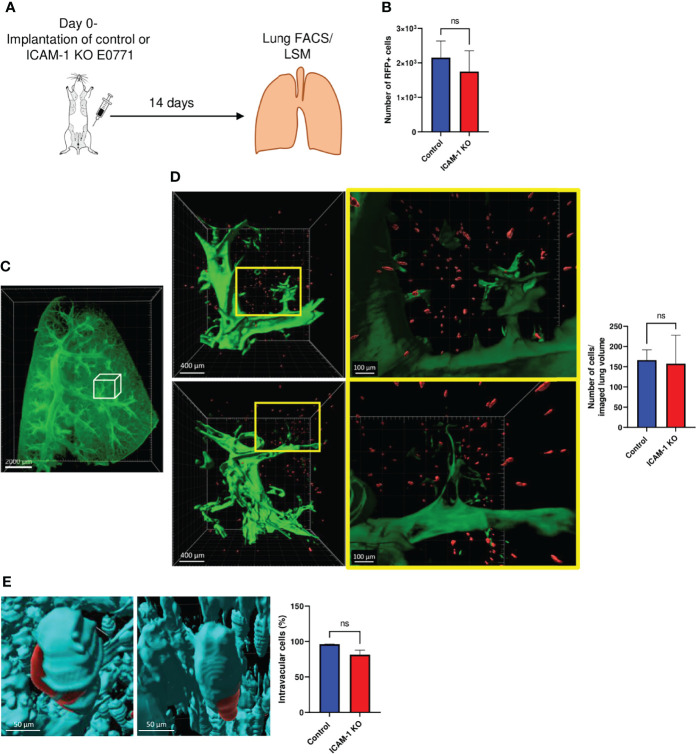
Dissemination and early accumulation inside the blood vessels of E0771 breast cancer cells are not affected by their ICAM-1 expression in a spontaneous model of metastasis to lungs. **(A)** A scheme depicting the experiment. 3 × 10^5^ control or ICAM-1 KO RFP-expressing E0771 cells were implanted in the mammary fat pads of recipient mice. Two weeks later, lungs were harvested and processed for either the FACS analysis of total cell suspensions or for the fixation and clearance of lungs for imaging by three-dimensional (3D) light sheet fluorescence microscopy (LSM). For imaging, mice were intravenously stained with Alexa fluor 647 labeled CD31 mAb injected i.v. 15 min before euthanasia and lung harvesting. **(B)** The number of RFP-positive E0771 cells recovered in the lungs determined by FACS. n=4. **(C)** A representative LSM image of a whole lung lobe taken from **Movie 8**. The white cube represents the lung volume (2,160 µm × 2,560 µm × 2,000 µm) imaged in D. **(D)** A representative LSM image of RFP cells disseminated from the primary breast tumor and accumulated in the lung lobes. Green: lung auto-fluorescence. Red: E0771 cells in the imaged cube depicted in C, representative of four lung lobes isolated from two mice. The right images are the magnifications of the yellow rectangles marked in the middle images. Top images: control E0771 cells. Bottom images: ICAM-1 KO E0771 cells. The spots representing the RFP signal were <3,000 µm^3^ in volume. The bars in the right panel depict the average number ± SEM of control and ICAM-1 KO E0771 cells (spots) detected in 4 lung cubes (2,160 µm × 2,560 µm × 2,000 µm in dimension). **(E)** The left and right images depict, respectively, individual control and ICAM-1 KO E0771 cells (red) and CD31-labeled lung vessels (cyan). Images were taken from the LSM-analyzed lung samples represented by the examples in **Movie 3**. The bars in the right panel depict the percentage of control or ICAM-1 KO E0771 cells detected inside the CD31-labeled lung vessels. The bars in **(B, D**, **E)** depict the mean values ± SEM of the same 4 lung cubes analyzed in D.

Advanced breast tumors consisting of cancer cells orthotopically implanted in syngeneic immunocompetent mice can give rise to spontaneous metastasis at various target organs. Since ICAM-1 deletion did not affect primary E0771 breast tumor growth, we next used a model of spontaneous metastasis established for E0771 (53) to test if the presence of ICAM-1 on orthotopically implanted E0771 cells affects their lung metastasis potential. To that end, we implanted large primary orthotopic tumors in recipient syngeneic mice, resected the tumors, and followed individual metastatic E0771 cells that have disseminated into the circulation either prior to or after resection and accumulated in the lungs. To accurately determine the number of accumulated E0771 cells and their precise location, we introduced into our control and ICAM-1 KO E0771 cell lines a red fluorescent protein (RFP) reporter. Importantly, as observed with small E0771 breast tumors (**Figure 3A**), the growth of the large tumors (**Supplementary Figure 5A**) and the main leukocyte composition of their TME (**Supplementary Figure 5B**) were unaffected by E0771 ICAM-1 expression. Nevertheless, we could not detect the circulating RFP+ tumor cells (CTCs) of either control or ICAM-1 KO E0771 cell tumors in the circulation following the resection of the respective primary breast tumors (data not shown). In contrast, another well-studied breast cancer line that metastasizes to lungs, 4T1, gave rise to spontaneous CTC generation in immunodeficient NSG mice as reported (39) (**Supplementary Figure 6B**), and all CTCs recovered from these mice expressed high levels of both ICAM-1 and the myeloid-specific chemokines we also found in E0771 cells (**Supplementary Figures 3, 6****, and** **Supplementary Table 1**). Nevertheless, 4T1 and E0771 varied considerably with respect to the expression of another key integrin ligand, the VLA-4 ligand VCAM-1. Whereas E0771 expressed only negligible levels of VCAM-1, 4T1 cells expressed significant levels of this VLA-4 ligand (**Supplementary Figures 6****)**. Interestingly, although we could not detect circulating RFP+ E0771 CTCs in the systemic circulation of E0771 tumor-bearing C57BL/6 mice, these cells were readily detected in the lungs of all mice implanted with large primary breast tumors (**Figures 4A, B****)**. Nevertheless, and in agreement with a recent study that suggests that E0771 cells do not metastasize to bones (53), we could not detect E0771 metastasis to this organ or to the liver (data not shown). Notably, similar numbers of both control and KO RFP–expressing E0771 cells were recovered in the total lung suspensions of recipient mice 14 days after their orthotopic implantation (**Figures 4A** **, B**). At this time point, similar numbers of control and ICAM-1 KO RFP-expressing E0771 cells were also detected by the three-dimensional (3D) imaging of whole lung lobes using the light sheet microscopy (LSM) of lipid-cleared lungs (**Figures 4C** **, D** and **Movie 1**). These results suggested that although E0771 CTCs could not be detected in our tumor implanted mice, similar numbers of control and ICAM-1 KO E0771 were most likely released from their respective primary tumors and accumulated in the lungs. Interestingly, the vast majority of the RFP+ E0771 cells detected in the lungs at this early time point were intravascular irrespective of their ICAM-1 expression (**Figure 4E** and **Movie 2**). These data collectively suggested that E0771 cells are released from primary breast tumors, enter the circulation, and subsequently get entrapped in the pulmonary vasculature independently of their ICAM-1 expression.

**Figure 5:**
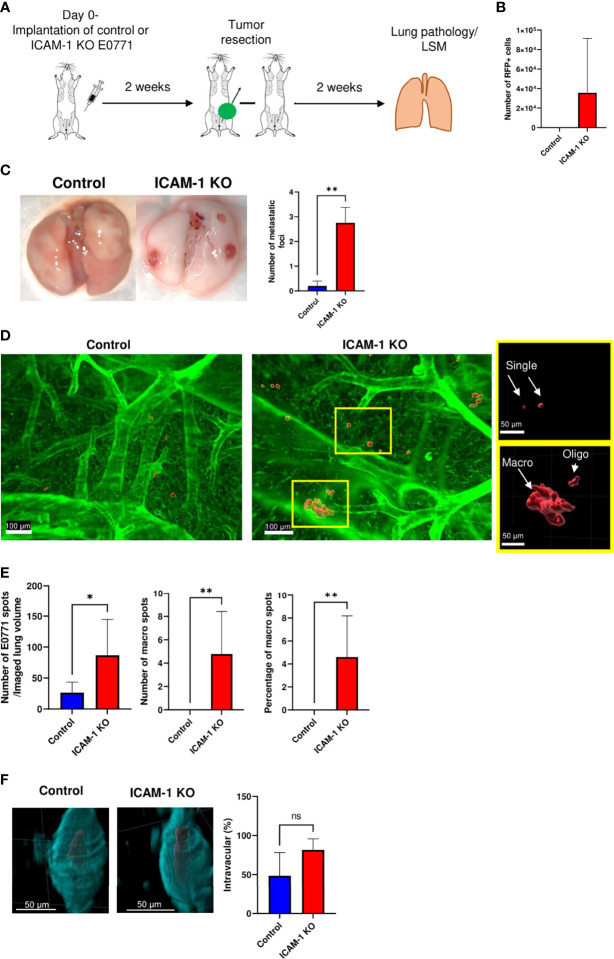
ICAM-1 expression by E0771 breast cancer cells suppresses the late growth of disseminated cancer cells in a spontaneous model of breast cancer metastasis to lung. **(A)** A scheme depicting the experiment. 3×10^5^ control or ICAM-1 KO E0771 cells were implanted in the mammary fat pads of recipient mice. Tumors were resected 2 weeks later, and 2 weeks after resection, the lungs were harvested and processed for either the FACS analysis of total cell suspensions or of lungs subjected to fixation and clearance for imaging by LSM. The mice were intravenously stained with Alexa fluor 647-labeled CD31 mAb 15 min before lung harvesting as in **Figure 4**. **(B)** The number of RFP-positive E0771 cells recovered in the lungs determined by FACS. The bars depict the mean values ± SEM n=6. **(C)** Two weeks after tumor resection, lungs were harvested and the number of tumor foci detected on the lung surface was determined. The left panel depicts representative lung images. The right panel depicts the number of tumor foci on the lung surface ± SEM. n=4. ​**p<0.005. **(D)** Representative LSM images of RFP control and ICAM-1 KO cells disseminated from their respective primary breast tumors and accumulated in the lung lobes. The rectangles in the ICAM-1 KO lung image contain representative single tumor cells (i.e., spots of <3,000 µm^3^, top rectangle) or either oligocellular lesions or macrolesions (>40,000 µm^3^, bottom rectangle). The rectangles are each magnified in the two right images. Green: lung auto-fluorescence. Red: E0771 cells. **(E)** The left graph depicts the mean ± SEM number of RFP E0771 spots detected in 8 imaged lung cubes (each 2,160 µm × 2,560 µm × 2,000 µm in dimension). The middle graph depicts the mean ± SEM number of macrolesions detected in 8 lung cubes. The right graph depicts the mean ± SEM percentages of macrolesions out of all E0771 spots detected. *p<0.05; **p<0.005. **(F)** The left and right images depict, respectively, control and ICAM-1 KO E0771 cells (red) entrapped inside CD31-labeled lung blood vessels (cyan). The bars in the right panel depict the percentage of control or ICAM-1 KO E0771 cell spots detected inside the CD31-labeled lung vessels. Results are mean ± SEM of the same 8 lung cubes analyzed in E.

Next, we followed the fate of these disseminated RFP+ E0771 cells recovered in recipient lungs 2 weeks after resection of the primary breast tumor (**Figure 5A**). Strikingly, at this time point, whereas the number of recovered control E0771 cells was reduced 15-fold from 2,150 ± 480 cells (**Figure 4B**, left bar) to 145 ± 80 cells (**Figure 5B**, left bar, *p*<0.0004), the number of ICAM-1 KO E0771 cells recovered from similar lung cell suspensions increased 20-fold, from 1,750 ± 600 (**Figure 4B**, left bar) to 35,000 ± 17,650 cells (**Figure 5B**, right bar, *p*<0.25). These results suggest that although the initial accumulation of ICAM-1 KO RFP+ E0771 cells inside the lung was normal, ICAM-1-deficient RFP+ E0771 cells were protected and readily expanded inside the lungs. We next repeated this line of experiments with non-RFP labeled E0771 cells and their ICAM-1 KO E0771 counterparts. Notably, and consistent with the FACS based results, resected ICAM-1 KO E0771 tumors gave rise to large metastatic nodules detected on the surface of the lungs while control E0771 did not (**Figure 5C**).

To further investigate the basis for the superior retention and survival of ICAM-1 KO RFP+ E0771 cells inside the recipient lungs, we next analyzed the distribution of these cells by the LSM of whole lung lobes. This analysis indicated that while control RFP+ E0771 cells remained singular (**Figure 5D**, left image), a fraction of ICAM-1 KO RFP+ E0771 cells gave rise to both oligoclusters and large macrocellular clusters (**Figure 5D**, right image and the two magnified rectangles, and **Movie 3**). Consistent with the FACS results (**Figure 5B**), both the total number of detectable RFP foci (i.e., spots of any dimension) and the fraction of macrocellular assemblies within these foci were significantly higher for ICAM-1 KO RFP-E0771 cells than for control RFP+ E0771 cells (**Figure 5E**). Strikingly, the LSM images indicated that the majority of ICAM-1 KO E0771 that survived and expanded in the lungs as singular cells or clusters remained inside the pulmonary vasculature, giving rise to intravascular metastasis (**Figure 5F** and **Movie 4**). These results collectively suggest that following the initial entry and accumulation of both control and ICAM-1 KO RFP+ E0771 cells inside the lung vasculature, ICAM-1-deficient E0771 cells, unlike their control counterparts, remain protected and can readily expand inside the pulmonary vasculature.

### Breast Cancer ICAM-1 Also Suppresses Experimental Metastasis to Lungs

**Figure 6:**
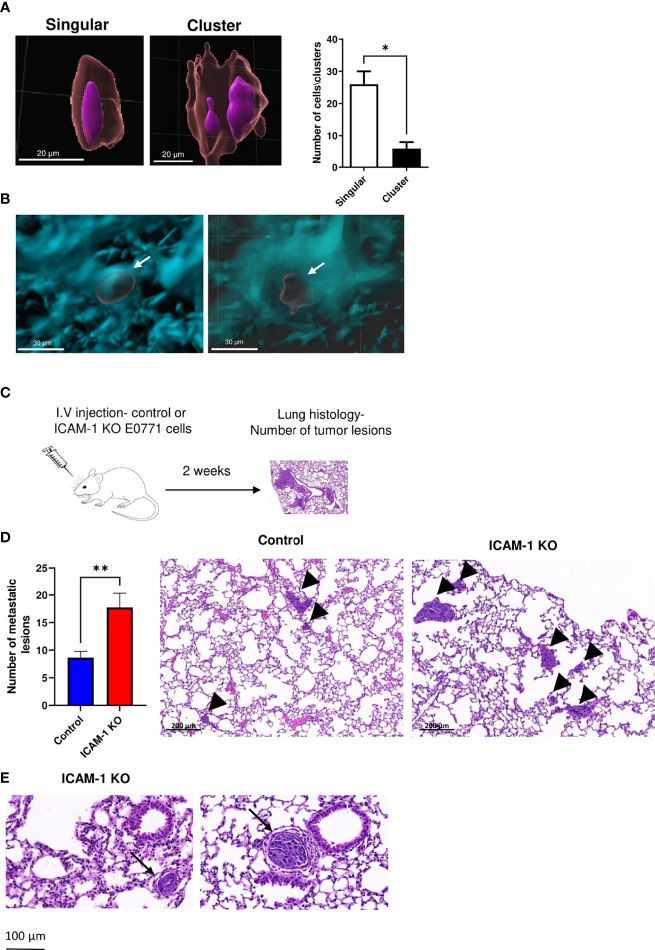
E0771 breast cancer ICAM-1 suppresses experimental metastasis to lung. **(A)** Visualization of singular and clustered E0771 cells accumulated inside lungs imaged by LSM. 1 × 10^4^ WT E0771 cells were injected i.v. Nuclei were labeled with REEDOT1. Nuclei (purple) and CMTMR-labeled E0771 breast cancer cells (red) were imaged by the LSM of cleared lungs lobes as outlined in **Figures 4**, **5**. Bars = 20 µm. The right panel depicts the number of single cells and clusters detected in each imaged lung cube as in **Figures 4**, **5**. The left lobes of two mice were imaged. The bars depict the mean values ± SEM. **(B)** LSM images of cleared lungs labeled with anti-CD31 mAb as in **Figures 4**, **5**, 1 h postinjection of 1 × 10^4^ CMTMR-labeled E0771 cells (left panel) or 2 weeks postinjection of 10^4^ RFP E0771 (right panel). **(C)** A scheme of the experimental metastasis model. Approximately 10^4^ control or ICAM-1 KO E0771 cells were injected i.v., and 14 days later, lungs were harvested and the number of E0771 lesions was determined by the H&E staining of 5-µm-thin lung sections. **(D)** Left panel: The number of metastatic lesions detected in 100 lung sections of 20 mice. A total of 5 lung sections 100 μm apart were isolated from the left lung lobe of each recipient mouse. The bars depict the mean values ± SEM. **p<0.005. The right panel depicts the representative histological sections of the two different experimental groups. The arrowheads depict either small or large lesions. **(E)** H&E staining of the representative lung sections of lung lobes containing ICAM-1 KO lesions. The arrows depict intravascular lesions in small (left) and large (right) vessels.

To further dissect the potential role of ICAM-1 in the suppression of the lung metastasis of intravascularly entrapped E0771 cells observed in the spontaneous metastasis model (**Figure 5**), we took a complementary approach, based on an experimental metastasis model of i.v.-injected E0771 cells. This model bypasses the steps involved in CTC dissemination from the primary tumor (i.e., intravasation into the circulation) but enables to directly investigate in defined time points and in a synchronized manner the potential communication of these cells with leukocytes circulating through the lung vasculature. Imaging control E0771 cells by LSM shortly after their i.v. injection into syngeneic C57BL/6 recipients indicated that all the cells reaching the lungs became entrapped inside CD31+ vessels primarily as singular cells (**Figures 6A** **, B**, and **Movie 5**) and could be detected inside these vessels even 14 days later (**Figure 6B** **and** **Movie 6**). Notably, we could not find any injected E0771 cells entering the lungs inside peribronchial blood vessels (**Supplementary Figure 7** and **Movie 7**), readily detected by their constitutive expression of the integrin ligand VCAM-1 (54). These findings indicated a preference of E0771 cells to arrest inside pulmonary capillaries. Furthermore, consistent with the spontaneous E0771 lung metastasis model, when the lungs of recipient mice injected with either control or ICAM-1 KO E0771 cells were harvested 14 days after i.v. injection and analyzed by H&E histology (**Figure 6C**), the number of metastatic lesions generated by ICAM-1 KO E0771 cells significantly exceeded those generated by control E0771 cells (**Figure 6D**), and metastatic ICAM-1 KO cell clusters could be detected inside rather than outside lung capillaries at this time point (**Figure 6E**).

**Figure 7:**
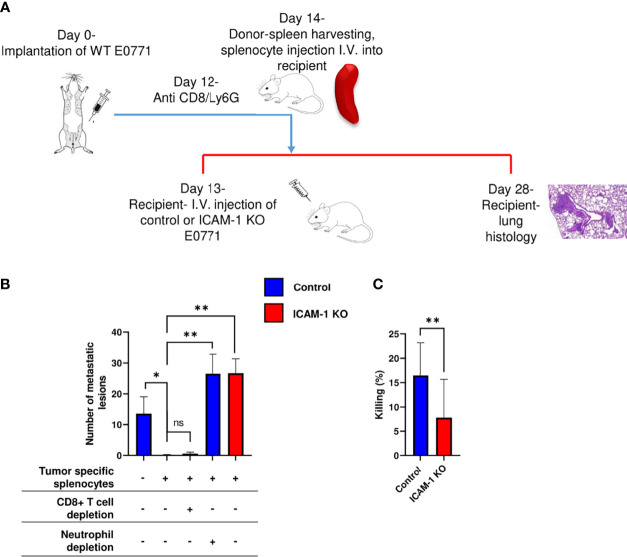
Adoptively transferred neutrophils eliminate E0771 cells entering the lungs *via* breast cancer expressed ICAM-1. **(A)** A scheme of the experimental outline. Metastatic lesions developed by either control or ICAM-1 KO E0771 cells injected into the lungs of recipient mice was determined in the mice pretreated with either naïve or tumor entrained splenocytes isolated from donor spleens and introduced i.v. 24 h after E0771 injection. At the indicated groups, CD8+ T cells or neutrophils were depleted in donor mice with an i.p. injection of anti CD8 or Ly6G, respectively, 2 days before the adoptive transfer**. (B)** The number of metastatic lesions developed in recipient lungs subjected to the indicated treatments determined by H&E staining as in previous figures. The first experimental group was transferred with naïve splenocytes. Each group consisted of 3 mice. *p<0.05. **p<0.005 NS = non-significant. **(C)** *In vitro* killing of control or ICAM-1 KO E0771 cells by the blood-derived neutrophils of tumor-bearing mice determined in triplicates. n=12. **p<0.005. The bars in B and C depict the mean values ± SEM.

Neutrophils recovered from breast tumor–bearing mice [termed tumor-entrained neutrophils (TENs)] readily eliminate lung-entrapped breast cancer cells like 4T1 cells (15). To assess the effects of similar circulating TENs on the metastatic potential of either control or ICAM-1 KO E0771 cells in our experimental system, we designed a protocol for the i.v. injection of control E0771 cells to syngeneic recipient mice followed by a transfer of spleen cell suspensions from control E0771 breast tumor–bearing donor mice to the recipient mice (**Figure 7A**). The transfer of a whole spleen was designed to avoid purifying TENs generated in the donor mice (**Figure 7A**). As reported, the donor mice implanted with orthotopic E0771 tumors exhibited massive neutrophilia and splenomegaly (15), but their content of inflammatory monocytes was unaltered (**Supplementary Figure 8**). Notably, the ability of lung vessel–entrapped E0771 breast cancer cells to generate metastatic lesions 2 weeks after injection was essentially eliminated by the donor splenocytes (**Figure 7B**, first vs. second bar). We next repeated this procedure, but instead of transferring intact donor splenocytes from donor E0771 tumor- bearing mice, we transferred the donor spleen cell suspensions of tumor-bearing donor mice that have been *in vivo* depleted of their neutrophils (by at least 90%) or CD8 T cells (by at least 95%, **Supplementary Figure 9**). Notably, while neutrophil-depleted splenocytes isolated from E0771 tumor-bearing donor mice could not eliminate the metastatic lesions of the lung vessel–entrapped E0771, CD8 T-cell-depleted splenocytes isolated from the donor mice normally eliminated all the metastatic lesions (**Figure 7B**, first vs. third or fourth bars, **Supplementary Figure 9**). Thus, neutrophils derived from the spleens of E0771 tumor-bearing donor mice but not CTLs derived from similar donor spleens can readily eliminate the metastatic lesions of control E0771 cells entrapped inside lung vessels. Furthermore, the ability of these donor spleen neutrophils to eliminate E0771 lung metastasis was entirely dependent on the presence of ICAM-1 on the breast cancer cells (**Figure 7B**, second vs. fifth bar). Thus, neutrophils accumulated in the spleens of tumor-bearing donor mice can readily enter the lung vasculature and use ICAM-1 on E0771 cells entrapped inside this vasculature for the elimination of the breast cancer targets. Consistent with these *in vivo* results, TENs isolated from E0771 breast tumor-bearing mice could also kill control E0771 cells more readily than their ICAM-1-deficient E0771 cell counterparts *in vitro* (**Figure 7C**). Collectively, our results suggest that TENs use ICAM-1 expression on E0771 cells entrapped inside the lung vasculature to eliminate intravascular metastatic breast cancer cells before they can expand into metastatic lesions.

## Discussion

ICAM-1 is expressed on the surface of several types of solid malignant cells (55, 56), as well as on essentially all hematopoietic cancer cells such as leukemia and lymphoma (55). As it is recognized by multiple β_2_ integrins expressed by all leukocytes, it could contribute to tumor growth by recruiting various tumor promoting and immune suppressory leukocytes to the TME, especially macrophages and neutrophils (57, 58). On the other hand, its presence could facilitate tumor elimination by different adaptive and non-adaptive immune cells (7, 59). To address these multiple potential functions of tumor-expressed ICAM-1, we have used the ICAM-1-expressing luminal B breast cancer line E0771 (21, 60) implanted in C57BL/6 immunocompetent syngeneic mice hosts as our model system. Our findings suggest that the growth of these breast cancer cells in primary orthotopic tumors is not affected by ICAM-1 deletion, whereas the ability of these breast cancer cells to give rise to metastatic lesions in the lungs is dramatically increased in the absence of ICAM-1.

The normal growth of ICAM-1-deficient breast cancer cells in primary tumors was associated with the normal tumor content of various CD45+ immune cells recruited by primary E0771 tumors with a small reduction in the DC and B-cell content in the draining LNs of the breast cancer tumors. Thus, although TAMs and DCs express, respectively, the ICAM-1 binding CD18 integrins, CD11b (57) and CD11C (α_x_β_2_) (61), their accumulation inside tumors did not require tumor-expressed ICAM-1, reflecting the potential usage by these cells of other Mac-1 and CD11C ligands expressed on cancer cells and in the TME including ECM components and proteoglycans (62, 63).

Adaptive immunity plays a pivotal role in tumor eradication, with the main activity attributed to tumor antigen–specific cytotoxic CD8+ T cells (CTLs) (64). In spite of their low immunogenicity, implanted E0771 were readily eliminated by endogenous antitumor T cells when endogenous Tregs were depleted (50). Furthermore, E0771 cells could elicit potent immunity toward identical tumors implanted in a distal mammary fat pad. Nevertheless, The elimination of the primary E0771 tumor by Treg depletion was not reduced when ICAM-1 was deleted on target E0771 cells. Similarly, the elimination of a primary E0771 tumor by a distally implanted homologous tumor also did not require the presence of ICAM-1 on E0771 cells. To further dissect the role of E0771 ICAM-1 in CTL-mediated killing, we have also bypassed these two endogenous pathways of CTL-mediated killing by targeting adoptively transferred effector OVA–specific CTLs into orthotopic E0771 tumors engineered to stably express OVA. In the presence of these exogenous CTLs, OVA-expressing E0771 tumors were readily eradicated by CTLs. Strikingly, however, *in vivo* OVA E0771 killing by CTLs did not also require the presence of ICAM-1 on the cancer cell targets. Collectively, these results suggest that the killing of E0771 cells within primary breast tumors by either endogenous or exogenous (i.e., OVA-specific) Ag-specific CTLs does not require ICAM-1 expression on the tumor cell targets. These results could not be explained by the function of alternative integrin ligands on E0771 breast cancer cells such as VCAM-1 (a VLA-4 ligand) or E-cadherin, a CD103 ligand expressed by certain solid tumor cells (65). E0771 cells do not express the E-cadherin and CTL-mediated killing of either control or ICAM-1 KO E0771 cells and could not be inhibited by VLA-4 blocking (data not shown). Notably, in spite of LFA-1 involvement in *in vitro* TIL interactions with the subsets of transformed B cells (17, 48), accumulating real-time microscopy studies suggest that stable CTL synapses are not a prerequisite for the killing of various antigen-presenting cells of a non-hematopoietic origin (66). Indeed, *in vivo* CTL interactions with virus-infected fibroblasts involve highly dynamic contacts between CTLs and the target cells characterized by sustained CTL motility on the target (67). Our present findings are therefore consistent with these observations, suggesting that ICAM-1 expression on breast cancer cells and possibly on other solid tumors is not obligatory for CTL-mediated killing.

The lung is the second most common site for the occurrence of metastasis after the bone (68). Tumors that originate from the breast, bladder, colon, kidney, head, neck, and skin (melanoma) all tend to metastasize to the lungs (69). The majority of studies on tumor metastasis to lungs have relied on the histology of thin sections or on the readouts of progressed metastasis, which seem inferior to the 3D-based LSM microscopy we have used in the present study. It was widely believed that cancer cells entrapped in small diameter vessels must extravasate and reside in interstitial spaces of the lungs in order to generate metastatic lesions (70). Using a new high-resolution 3D imaging approach, we found both in the experimental and spontaneous models of E0771 metastasis in the lungs that a major fraction of the breast cancer cells that got entrapped in the large pulmonary vascular bed survived inside these blood vessels and gave rise to intravascular hematogenous metastasis. So far, the ability of CTCs to give rise to such type of metastasis has been shown only in xenograft models (71, 72). Our results indicate for the first time that metastatic breast cancer cells can also generate intravascular lung metastasis in syngeneic immunocompetent mice. Although the subclones of E0771 and other breast cancer cells that express ICAM-1 like 4T1 can establish extravascular metastasis in immunocompetent mice (26, 73), it appears that the parental E0771 cells we have used represent a major subgroup of breast tumor–derived CTCs capable of growing inside the lung vasculature (71). The molecular basis for the preference of E0771 cells to give rise to intravascular metastasis is unclear. E0771 cells might be deficient in functional factors that normally increase vascular hyperpermeability and are presumably critical for cancer cell extravasation across the lung capillaries. Indeed, factors like angiopoietin-like-4 (Angptl4), VEGF, COX-2, and a variety of proteases, including MMP-1, and MMP-2, and MMP-9 (70, 74) are transcribed at low levels by E0771 cells (41).

Our results suggest that the survival of breast cancer cells like E0771 cells inside the lung vasculature likely requires these cancer cells to evade killing by circulating killer neutrophils enriched inside mice with primary breast tumors. These cytotoxic neutrophils termed tumor-entrained neutrophils (TENs) were originally identified in breast cancer–bearing mice (15). Our current findings suggest for the first time that breast cancer–expressed ICAM-1 accounts for the susceptibility of breast cancer CTCs entrapped inside lung vessels to intravascular killing by these neutrophils, most probably as they circulate through these vessels. Neutrophils are the major type of WBCs in the circulation (75). The diversity of neutrophils recruited into primary tumors or metastatic lesions at various organs and their modes of activity has been well recognized (15, 39, 76–79). The contributions of distinct subsets of neutrophils to carcinogenesis and metastatic processes vary, as both protumor and antitumor activities were observed in different models and organs (80). Consequently, the time window of a leukocyte-neutralizing antibody injection often results in different outcomes of tumor progression or suppression (81). The TEN subset involved in E0771 elimination inside the lung vasculature most probably consists of cytotoxic neutrophils that release H_2_O_2_ and target the H_2_O_2_-dependent Ca^2+^ channel TRPM2 (82) on breast cancer cells (83). We have verified that both control and ICAM-1 KO E0771 cells express comparable levels of this channel (data not shown), ruling out the possibility that the reduced neutrophil-mediated killing of ICAM-1 KO E0771 cells is due to reduced TRPM2-mediated sensitivity to H_2_O_2_. However, the direct involvement of TRPM2 in E0771 cell killing by TENs remains to be demonstrated.

Our results link for the first time between neutrophil-mediated intravascular killing and the reduced ability of WT E0771 cells to give rise to intravascular lung metastasis. We suggest that capillary-entrapped WT E0771 cells most probably undergo selection in order to escape TEN-mediated killing. It is also possible that breast CTCs that downregulate TRPM2 or ICAM-1 expression [e.g., transcriptionally or by proteolytic shedding *via* the TACE/ADAM17 metalloprotease (80)] may give rise to more frequent intravascular metastasis, a possibility that needs further investigation in follow-up studies. In addition, recent works predict that distinct breast CTCs can interact not only with neutrophils (15, 77) but also with NK cells (26), distinct subsets of monocytes (84), intravascular macrophage subsets (85), and megakaryocytes (86). The outcome of these interactions is determined by a complex repertoire of tumor-expressed molecules, including “don’t eat me signals” (87, 88), integrin ligands, NK-activating ligands (26), and intracellular tumor expressed chemokines (89). Although not we did not genetically manipulate it, it is possible that E0771-expressed CXCL1 contributes to ICAM-1-dependent neutrophil–CTC adhesion and killing (90). It is also anticipated that the CTCs entrapped inside the lung capillaries are prone to proapoptotic mechanical stress (91) and must get protected in order to survive and proliferate inside the pulmonary capillaries (42, 92, 93). Notably, E0771 cells readily bind platelets (94) that may protect these cells from both leukocyte-driven and shear stress–induced apoptosis and provide the CTCs with prometastatic cytokines such as TGF-β (95). Future studies should shed more light on how distinct cancer-expressed integrin ligands mediate breast cancer cell communications with platelets and the different leukocytes marginated in the pulmonary vasculature (96). This information is necessary to design new blockers to interfere with cancer metastasis to lungs and other highly vascularized target organs.

## Data Availability Statement

Publicly available datasets were analyzed in this study. This data can be found here: https://www.jianguoyun.com/c/sd/145e7ae/4527c204757bb261#from=https%3A%2F%2Fwww.jianguoyun.com%2Fc%2Fsd%2F145e7ae%2F4527c204757bb261.

## Ethics Statement

The animal study was reviewed and approved by the Animal Care and Use Committee of the Weizmann Institute of Science.

## Author Contributions

OR designed and performed a major part of the *in vitro* and *in vivo* experiments, analyzed the data, and contributed to the writing of the manuscript. OY performed *in vitro* killing experiments. MK, SK, and NL assisted with *in vivo* experiments. FR conducted *in vitro* adhesion experiments. MS performed IF staining of CTCs. FC-G performed computational analysis of CTC transcriptome data. SB-D designed the CRISPR tools. YA assisted with data acquisition of LSM. OG assisted with data analysis of LSM images. MD performed the TCGA analysis. NA supervised the CTC experiments and revised parts of the manuscript. ZG supervised the *in vitro* killing experiments. RA supervised all the experiments and wrote the manuscript. All authors contributed to the article and approved the submitted version.

## Funding

RA is the incumbent of the Linda Jacobs Chair in Immune and Stem Cell Research. His research is supported by the Israel Science Foundation (grant no. 791/17), the Minerva Foundation, Germany, GIF (grant number I-1470-412.13/2018), Israel Cancer Research Fund (19-109-PG), EU Horizon 2020 Research and Innovation Program (Ri-boMed 857119), and grants from the Moross Integrated Cancer Center, Helen and Martin Kimmel Institute for Stem Cell Research, Meyer Henri Cancer Endowment, and from William and Marika Glied and Carol A. Milett. ZG is supported by grants from Israel Science Foundation (Grant No. 405/18), Israel Cancer Association, the Rosetrees Trust, and the Israel Cancer Research Foundation. Research in the Aceto lab is supported by the European Research Council (678834 and 840636), the European Union (801159-B2B), the Swiss National Science Foundation (PP0P3_163938, PP00P3_190077, IZLIZ3_182962), the Swiss Cancer League (KFS-3811-02-2016, KLS-4222-08-2017, KLS-4834-08-2019), the Basel Cancer League (KLbB-4173-03-2017, KLbB-4763-02-2019), the two Cantons of Basel through the ETH Zürich (PMB-01-16), and the University of Basel, Switzerland.

## Conflict of Interest

NA is a paid consultant for companies with an interest in liquid biopsy.

The remaining authors declare that the research was conducted in the absence of any commercial or financial relationships that could be construed as a potential conflict of interest.

## Publisher’s Note

All claims expressed in this article are solely those of the authors and do not necessarily represent those of their affiliated organizations, or those of the publisher, the editors and the reviewers. Any product that may be evaluated in this article, or claim that may be made by its manufacturer, is not guaranteed or endorsed by the publisher.

## Supplementary Material

Supplementary Table 1 Expression levels of selected genes in single 4T1 CTC (n=4), CTC clusters (n=4) and CTC-neutrophil clusters (n=6) derived from orthotopic breast cancer lesions generated in 8–10-week-old female NSG females upon the injection with 0.5 × 106 4T1-GFP cells into the mammary fat pad. Data was obtained from Gene Expression Omnibus (NCBI; accession number GSE109761) and expression levels were defined as log2 of Transcripts Per Kilobase Million (TPM). *P* values were determined by Kruskal–Wallis test.
