# Supplementary material for: ICAM-1 on Breast Cancer Cells Suppresses Lung Metastasis but Is Dispensable for Tumor Growth and Killing by Cytotoxic T Cells
Source: Front Immunol. 2022 Jul 11;13:849701. doi: 10.3389/fimmu.2022.849701 (PMC9328178; doi:10.3389/fimmu.2022.849701)
Supplement: Supplementary file 1 [file DataSheet_1.pdf]

Movie legends

Movie 1: LSM of control and ICAM-1 KO RFP expressing E0771 cells released from primary tumors and accumulated in the lungs in a spontaneous breast cancer metastasis model. Images were taken 2 weeks after the original implantation of these cells in the mammary fat pads.

Movie 2: LSM of control and ICAM-1 KO RFP expressing E0771 cells (red) released from primary tumors and accumulated in the lungs in a spontaneous breast cancer metastasis model 2 weeks after implantation in the mammary fat pads. Shortly before lung harvesting the mice were i.v. injected with Alexa-647 labeled anti murine CD31 in order to determine the location of E0771 cells relative to the lung vasculature (cyan).

Movie 3: LSM of ICAM-1 KO RFP expressing E0771 cells (red) surviving and expanding in the lungs in a spontaneous breast cancer metastasis. Images were taken 2 weeks after resection of the corresponding primary tumor. Green- auto fluorescence. Singular E0771 cells, small micro-clusters of E0771 cells and large macro-clusters are labeled with arrows. The experimental protocol is outlined in Figure 6A.

Movie 4: LSM of a macro-cluster of ICAM-1 KO RFP expressing E0771 cells (red) generated in the lungs during spontaneous breast cancer metastasis. Images were taken 2 weeks after resection of the corresponding primary tumor. Shortly before lung harvesting the mice were i.v. injected with Alexa-647 labeled anti murine CD31 in order to determine the location of the E0771 macro-cluster relative to the lung vasculature (cyan).

Movie 5: LSM of recipient lungs isolated 1 hr after i.v. injection of CMTMR-labeled E0771 cells (red). Lung vessels were stained by i.v. injection of Alexa-647 labeled anti-CD31 (cyan) shortly before lung harvesting.

Movie 6: LSM of recipient lungs isolated 14 days after i.v. injection of RFP E0771 cells (red). Lung vessels were stained by i.v. injection of Alexa-647 labeled anti-CD31 (cyan) shortly before lung harvesting.

Movie 7: LSM of recipient lungs isolated 1 hr after i.v. injection of CMTMR-labeled E0771 cells (red). Lung vessels were stained by i.v. injection of Alexa-647 labeled anti-VCAM-1 (cyan) shortly before lung harvesting.

Movie 8: LSM of a whole lung left lobe. The different airways are depicted by their auto fluorescence (green). A representative cubic volume used for subsequent imaging of individual cancer cells accumulated in the lungs is enlarged.
