# Supplementary material for: ICAM-1 on Breast Cancer Cells Suppresses Lung Metastasis but Is Dispensable for Tumor Growth and Killing by Cytotoxic T Cells
Source: Front Immunol. 2022 Jul 11;13:849701. doi: 10.3389/fimmu.2022.849701 (PMC9328178; doi:10.3389/fimmu.2022.849701)
Supplement: Supplementary file 2 [file DataSheet_2.pdf]

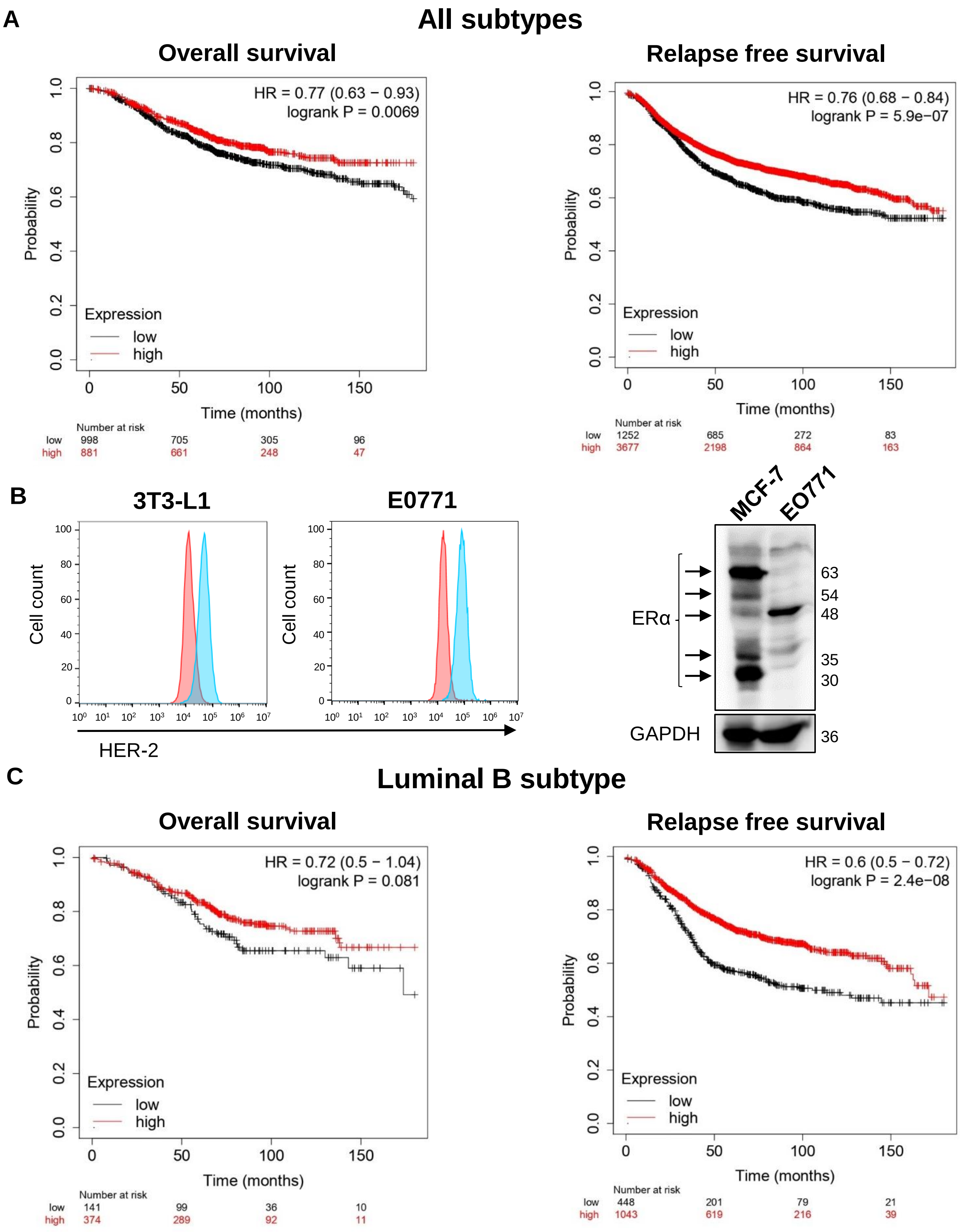

Supplementary Figure 1: High ICAM-1 expression in breast cancer is significantly associated with favorable outcome of breast cancer patients. (A) The correlation between ICAM-1 level and overall/relapse free survival of all subtypes breast cancer patients was evaluated based on the transcriptome level gene expression datasets, using the online tool Kaplan-Meier Plotter (https://kmplot.com/analysis/). (B) Left panel: The expression of HER-2 on 3T3-L1 (positive control) and E0771 cells was analyzed by FACS. Red- Secondary Ab only. Blue- anti-HER-2 + secondary Ab. Right panel: Equal amounts of proteins from MCF-7 (positive control) and E0771 cells, separated by SDS-PAGE and analyzed for ER-α by Western blot analysis. The cell lines display different isoforms of ER-α. GAPDH was used as a loading control. Arrows depict the dominant isoform. (C) The correlation between ICAM-1 level and overall/relapse free survival of Luminal B breast cancer patients was evaluated as in A.

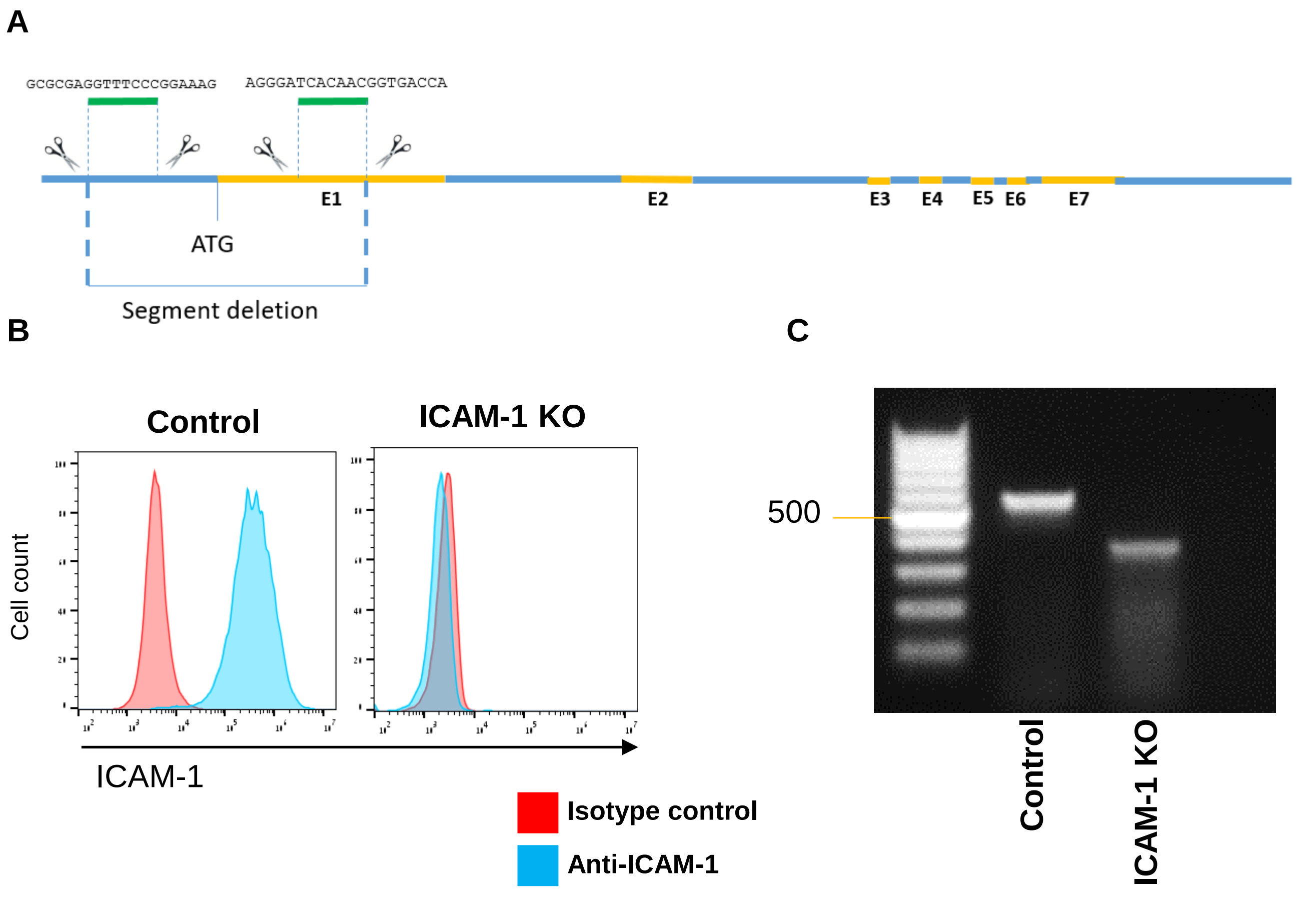

Supplementary Figure 2: ICAM-1 deletion in E0771 cells. (A) Design of ICAM-1 KO strategy in E0771 cells. ICAM-1 was knocked out using CRISPR-Cas9 upstream and downstream to the initiator methionine in the first exon. A 178 bp segment which includes the initiator methionine was selectively deleted. Guide sequences are shown above each deletion site. **(B)** Cells were sorted by FACS for ICAM-1 positive (Control) or ICAM-1 negative (ICAM-1 KO) cells. **(C)** The deletion of the 178 bp segment was verified by PCR, using primers upstream and downstream to the segment.

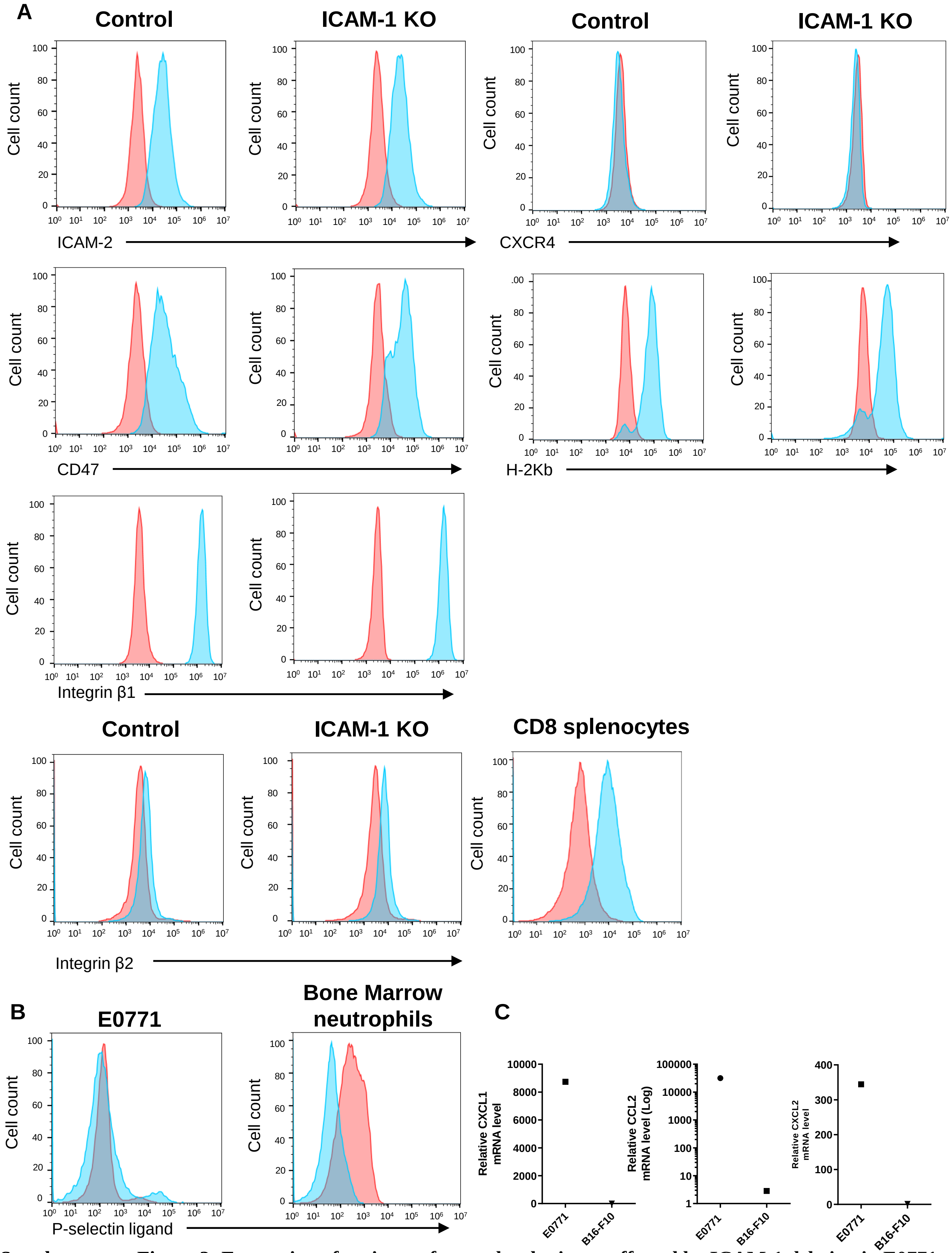

Supplementary Figure 3: Expression of major surface molecules is not affected by ICAM-1 deletion in E0771 cells. (A) Expression of the indicated surface molecules was analyzed by FACs. Each of the indicated markers was stained with directly labeled mAbs. Blue: specific mAb. Red: isotype control. **(B)** P-selectin ligand levels were determined with P-selectin-IgG fusion molecule as described in the materials and method section. Blue: specific mAb. Red: isotype control. **(C)** Relative transcription levels of the indicated chemokines in E0771 and B16-F10 cells analyzed by real time PCR. Values are normalized with β-actin mRNA content of each cell type.

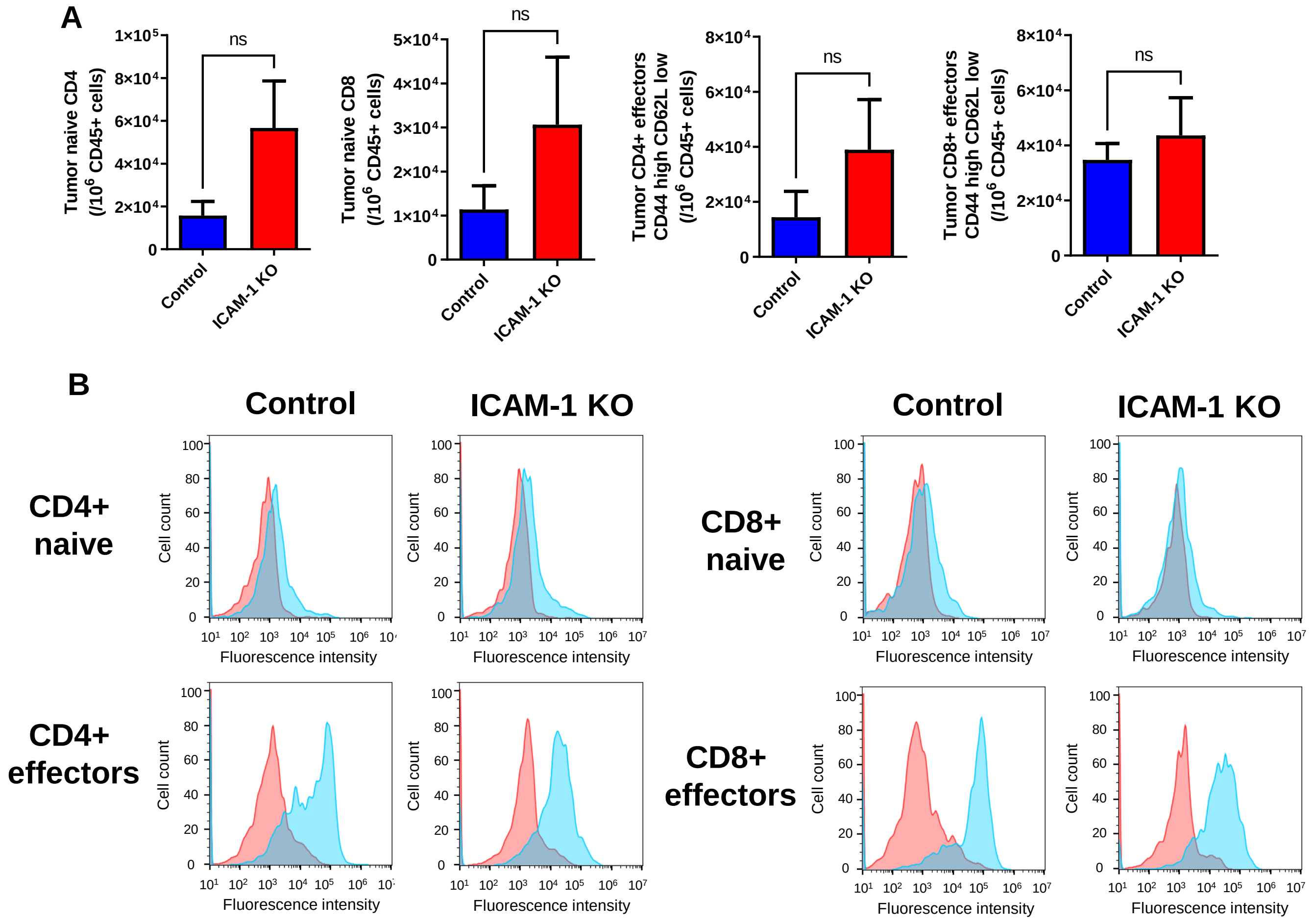

Supplementary Figure 4: Accumulation of effector T cells in the TME and their PD-1 expression are unaffected by the absence of E0771-expressed ICAM-1. $1x10^3$ control or ICAM-1 KO E0771 cells were implanted in the mammary fat pad of C57BL/6 female mice. 10 days later, tumors were harvested and single cell suspensions were prepared. **(A)** The numbers ± SEM of naïve ($CD44^{lo}$ $CD62L^{hi}$) and effector ($CD44^{hi}$ $CD62L^{lo}$) CD4+ and CD8+ T cells recovered in the tumor derived cell suspensions was determined by FACS. n=7. ns- non significant. The number of each subset was normalized to tumor weight. **(B)** PD-1 expression on the indicated groups of naïve or effector T cells was determined by FACS. Blue- Anti PD-1 mAb. Red- Isotype control.

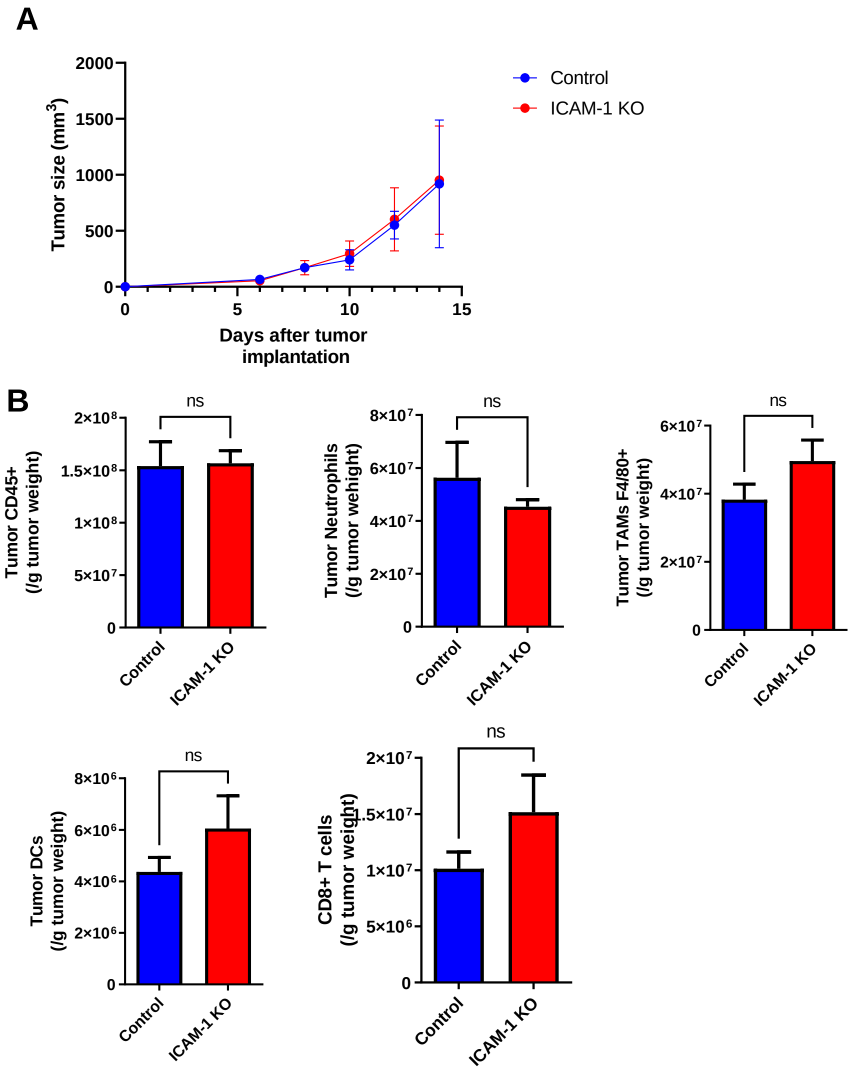

Supplementary Figure 5: Tumor growth and accumulation of different leukocyte subsets in the tumor microenvironment are not affected by the absence of ICAM-1. (A) $3x10^5$ control or ICAM-1 KO E0771 cells were suspended in Matrigel and implanted in the mammary fat pad of C57BL\6 female mice. Tumor size was measured every 2 days between day 6 and day 14. n=4. **(B)** Tumor cells were implanted as in A. 14 days later, tumors were harvested and single cell suspensions were prepared. The number ± SEM of CD45+ cells, neutrophils (CD45+, Ly6G^{hi}, CD11b^{hi}), TAMs (CD45+, F4/80+) and DCs (CD45+, MHCII^{hi}, CD11c^{hi}), recovered from the tumors was determined by FACs. The number of each leukocyte subset was normalized to tumor weight. n=4.

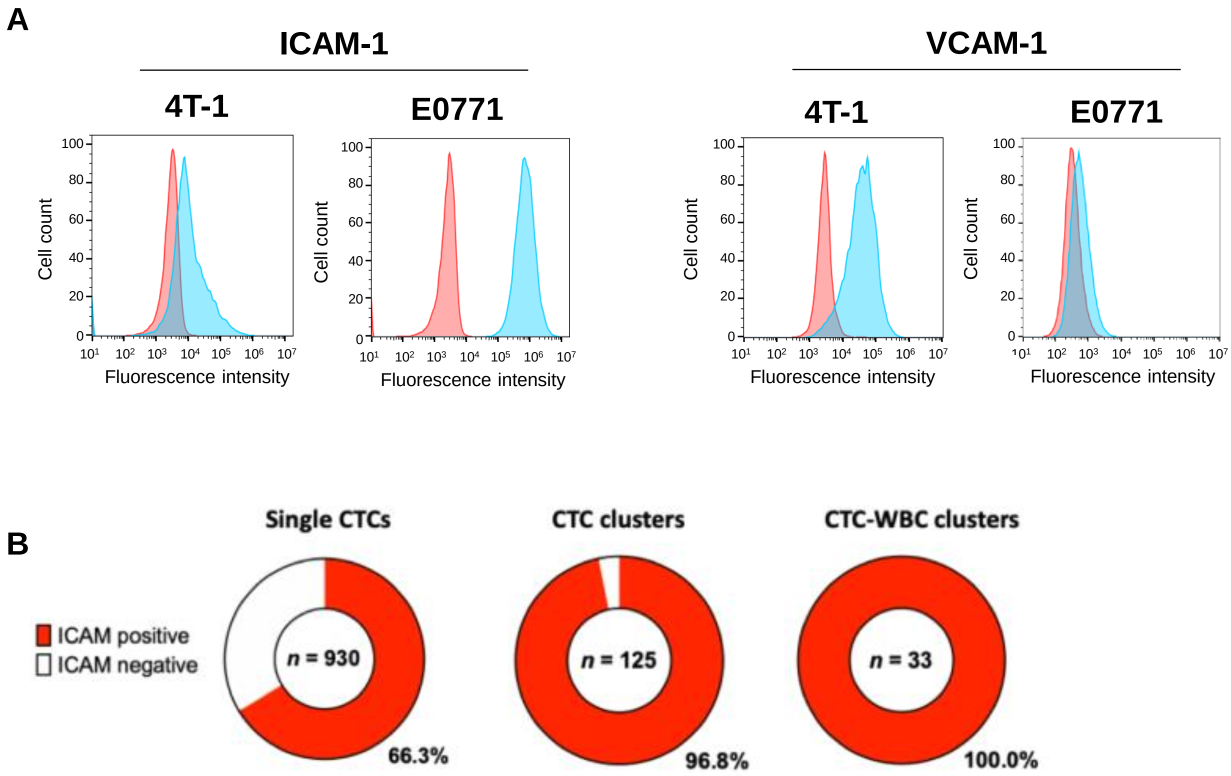

Supplementary Fig. 6: ICAM-1 expression is shared among different breast cancer cell lines. (A) ICAM-1 and VCAM-1 surface expression on 4T-1 and E0771 cell lines was determined with a directly labeled mAb (blue) and background staining was determined with an isotype match control mAb (red). **(B)** Quantification of ICAM-1-positive and negative species of circulating tumor cells (CTCs) in the peripheral blood of tumor bearing NSG mice transplanted with a primary 4T1-GFP breast tumor. Circulating cells were collected 3 weeks post transplantation. Pie charts display the mean percentage of ICAM-1 positive single CTCs, multicellular CTC clusters (homotypic) and CTC-WBC clusters (heterotypic) containing at least one WBC associated (arrow). The cumulative number of the indicated CTCs or CTC cluster species is shown for each chart, and it combines 3 replicates.

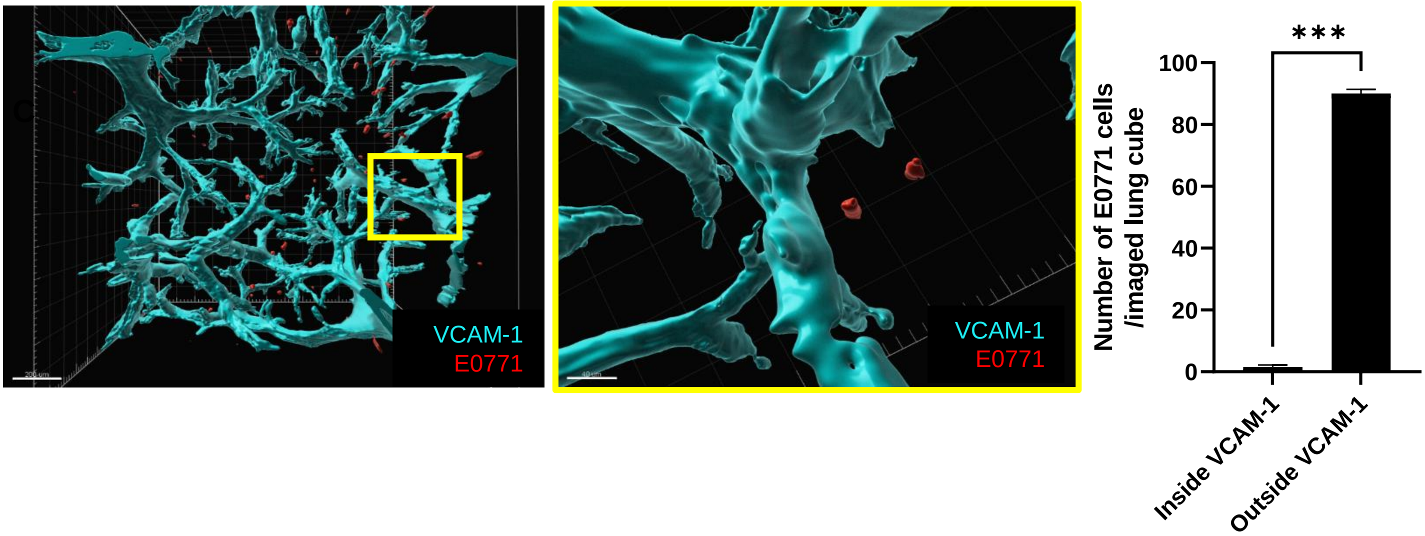

Supplementary Figure 7: E0771 breast cancer cells entering the lungs are not entrapped inside large VCAM-1 vessels. LSM based visualization of left lung lobes in mice injected i.v. with CMTMR labeled E0771 cells (red) and an anti VCAM-1 mAb (Cyan). 10^4 E0771 cells were injected i.v into recipient mice. 1 hour later, Alexa fluor 647 labeled anti VCAM-1 mAb was injected i.v. 15 minutes later mice were sacrificed and lungs were processed for LSM imaging. The images are representative of the two mice. Low and high magnification images of the same lung area are depicted. Left image bar= 200µm; right image bar= 40µm. 3D analysis of the same lung compartment is depicted in Movie 7. The numbers ± SEM of CMTMR labeled E0771 cells entering the lung entrapped inside or away from VCAM-1 expressing vessels are depicted in the bar graph below. ****p*<0.0005.

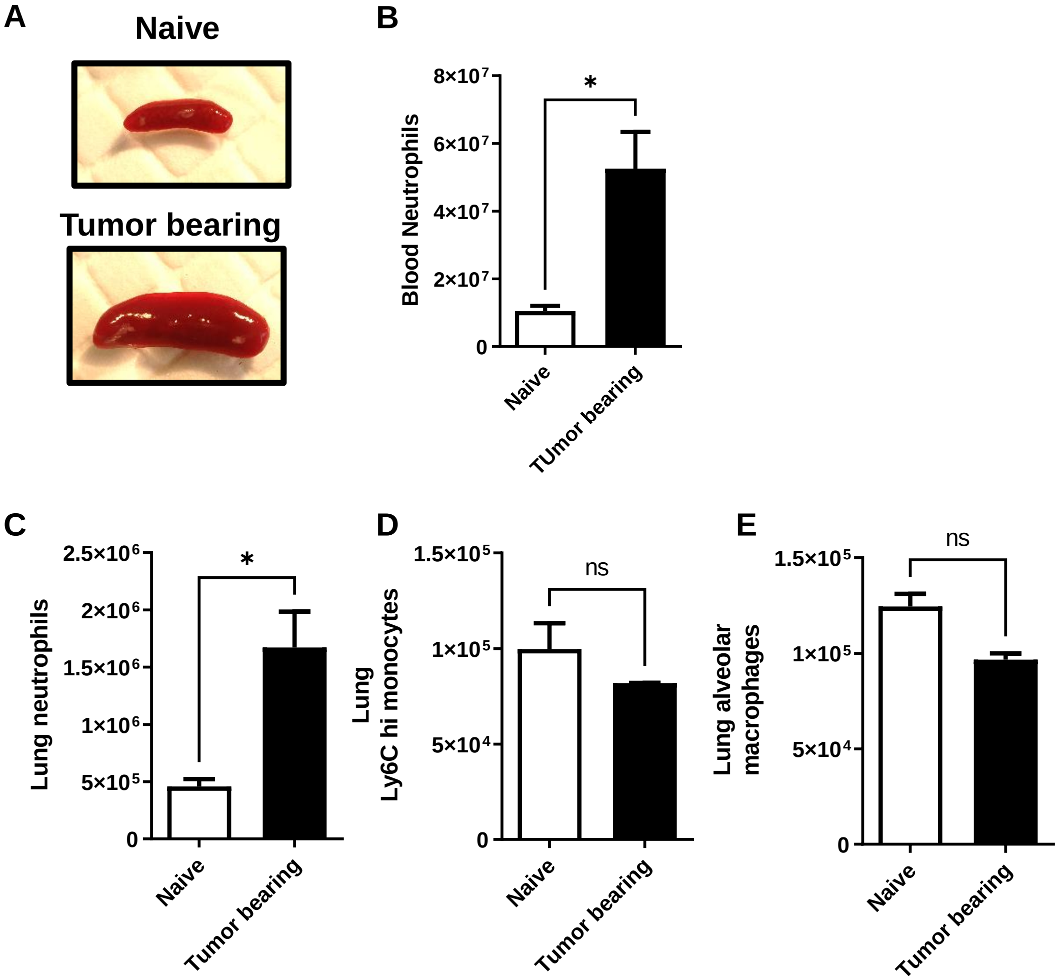

Supplementary Figure 8: A primary E0771 tumor induces major changes in the blood, spleen and lungs 2 weeks after implantation. **(A)** Spleens were harvested and imaged 4 weeks after E0771 implantation. **(B-E)** The number ± SEM of blood neutrophils (B), as well as lung neutrophils (C), lung Ly6C^{hi} (inflammatory) monocytes (D) and alveolar macrophages (E), were compared in naïve and tumor bearing mice by FACS two weeks after implantation of $1x10^3$ E0771 cells into the mammary fat pad of C57BL/6 female mice. n=3. Values are the mean +/- SEM. *p<0.05.

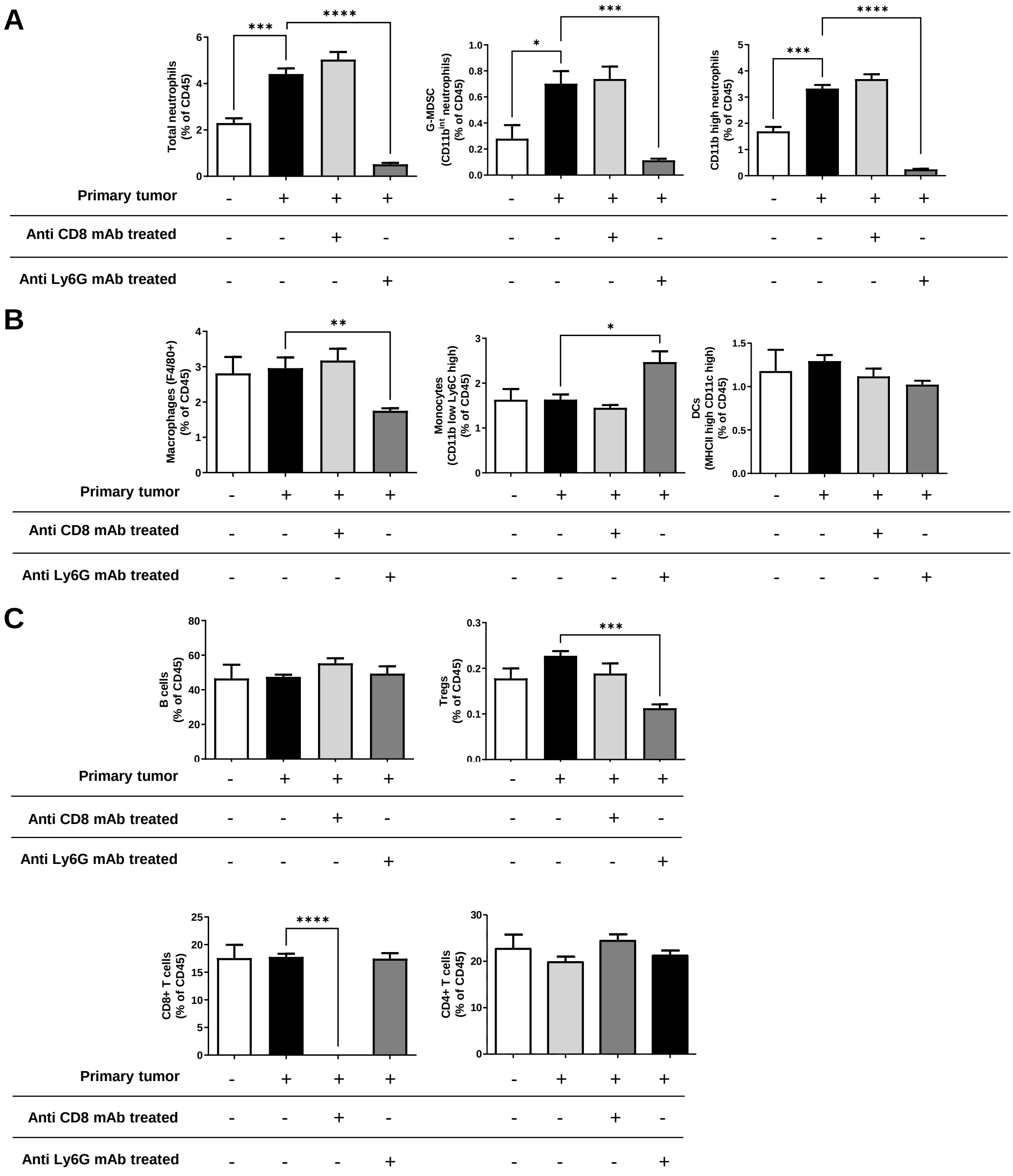

Supplementary Figure 9: Immune populations in the spleen of tumor bearing mice. 10^3 WT E0771 cells were suspended in Matrigel and implanted in the mammary fat pad of C57BL\6 female mice. 12 days later, where indicated, mice were treated with either anti CD8 mAb or anti Ly6G mAb as described in the Materials and Methods section. 2 days later, spleens were harvested and single cell suspensions were prepared. The numbers +/- SEM of (A) total neutrophils (CD45+, $Ly6G^{hi}$, $CD11b^{hi/int}$), G-MDSCs ($CD11b^{int}$ neutrophils) and $CD11b^{high}$ neutrophils, (B) macrophages (CD45+,F4/80+) inflammatory monocytes (CD45+, $CD11b^{low}$, $Ly6C^{hi}$), DCs (CD45+, $MHCII^{hi}$, $CD11c^{hi}$) and (C) B cells (CD45+, CD19+), Tregs (CD45+, CD4+, CD25+, FOXP3+), CD8+ T cells and CD4+ T cells recovered from the spleens were determined by FACs. Number of each leukocyte subset was normalized to total counts of spleen CD45+ cells. n=4. *p<0.05 **p<0.005 ***p<0.0005 ****p<0.0001.
