# Supplementary material for: ICAM-1 on Breast Cancer Cells Suppresses Lung Metastasis but Is Dispensable for Tumor Growth and Killing by Cytotoxic T Cells
Source: Front Immunol. 2022 Jul 11;13:849701. doi: 10.3389/fimmu.2022.849701 (PMC9328178; doi:10.3389/fimmu.2022.849701)
Supplement: Supplementary Table 1 — Expression levels of selected genes in single 4T1 CTC (n=4), CTC clusters (n=4) and CTC-neutrophil clusters (n=6) derived from orthotopic breast cancer lesions generated in 8–10-week-old female NSG females upon the injection with 0.5 × 10^6^ 4T1-GFP cells into the mammary fat pad. Data was obtained from Gene Expression Omnibus (NCBI; accession number GSE109761) and expression levels were defined as log2 of Transcripts Per Kilobase Million (TPM). P values were determined by Kruskal–Wallis test. [file Table_1.pdf]

		CTC Single (n=4)	CTC cluster (n=4)	CTC neutrophil (n=6)	p value
ICAM-1	Mean (SD)	5.89 (1.60)	3.48 (2.54)	4.39 (2.65)	0.52
	Median (Q1, Q3)	5.41 (4.82, 6.49)	4.14 (2.41, 5.21)	5.08 (3.24, 5.88)	
	Range	4.61-8.13	0.00-5.65	0.00-7.43	
CXCL1	Mean (SD)	7.81 (5.07)	7.72 (3.66)	10.41 (1.12)	0.644
	Median (Q1, Q3)	8.13 (4.02, 11.92)	6.90 (5.91, 8.71)	10.31 (9.59,11.24)	
	Range	2.44-12.53	4.23-12.86	9.11-11.85	
CXCL2	Mean (SD)	5.49 (6.36)	0.52 (1.04)	0.00 (0.00)	0.155
	Median (Q1, Q3)	5.15 (0.00, 10.64)	0.00 (0.00, 0.52)	0.00 (0.00-0.00)	
	Range	0.00-11.66	0.00-2.09	0.00-0.00	
CCL2	Mean (SD)	5.82 (5.58)	0.00 (0.00)	0.75 (1.83)	0.034
	Median (Q1, Q3)	4.93 (3.57, 7.17)	0.00 (0.00, 0.00)	0.00 (0.00, 0.00)	
	Range	0.00-13.42	0.00-0.00	0.00-4.49	
CD47	Mean (SD)	9.07 (0.62)	9.36 (0.28)	9.18 (0.30)	0.651
	Median (Q1, Q3)	9.21 (8.72,9.56)	9.24 (9.21, 9.39)	9.18 (8.92, 9.44)	
	Range	8.28-9.58	9.16-9.78	8.84-9.51	

Supplementary Table 1. Expression levels of selected genes in single 4T1 CTC (n=4), CTC clusters (n=4) and CTC-neutrophil clusters (n=6) derived from orthotopic breast cancer lesions generated in 8–10-week-old female NSG females upon the injection with 0.5 × 106 4T1-GFP cells into the mammary fat pad. Data was obtained from Gene Expression Omnibus (GEO, NCBI; accession number GSE109761) and expression levels were defined as log2 of Transcripts Per Kilobase Million (TPM). *P* values were determined by Kruskal–Wallis test.
